# Genome-wide identification and expression analysis of LBD transcription factor genes in Moso bamboo (*Phyllostachys edulis*)

**DOI:** 10.1186/s12870-021-03078-3

**Published:** 2021-06-28

**Authors:** Bin Huang, Zhinuo Huang, Ruifang Ma, Muthusamy Ramakrishnan, Jialu Chen, Zhijun Zhang, Kim Yrjälä

**Affiliations:** 1 State Key Laboratory of Subtropical Silviculture, Zhejiang A&F University, Lin’an, Hangzhou, 311300 Zhejiang Province People’s Republic of China; 2 Zhejiang Provincial Collaborative Innovation Centre for Bamboo Resources and High efficiency Utilization, Zhejiang A&F University, Zhejiang, China; 3 Co-Innovation Center for Sustainable Forestry in Southern China, Nanjing Forestry University, Nanjing, 210037 Jiangsu China; 4 Bamboo Research Institute, Nanjing Forestry University, Nanjing, 210037 Jiangsu China; 5 Department of Forest Sciences, University of Helsinki, Helsinki, Finland

**Keywords:** Moso bamboo, *LBD* gene family, Synteny analysis, Expression pattern, Target genes

## Abstract

**Background:**

Moso bamboo, the fastest growing plant on earth, is an important source for income in large areas of Asia, mainly cultivated in China. Lateral organ boundaries domain (LBD) proteins, a family of transcription factors unique to plants, are involved in multiple transcriptional regulatory pathways and play important roles in lateral organ development, pathogen response, secondary growth, and hormone response. The *LBD* gene family has not previously been characterized in moso bamboo (*Phyllostachys edulis*).

**Results:**

In this study, we identified 55 members of the *LBD* gene family from moso bamboo and found that they were distributed non-uniformly across its 18 chromosomes. Phylogenetic analysis showed that the moso bamboo *LBD* genes could be divided into two classes. *LBD*s from the same class share relatively conserved gene structures and sequences encoding similar amino acids. A large number of hormone response–associated *cis-*regulatory elements were identified in the *LBD* upstream promoter sequences. Synteny analysis indicated that *LBD*s in the moso bamboo genome showed greater collinearity with those of *O. sativa* (rice) and *Zea mays* (maize) than with those of Arabidopsis and *Capsicum annuum* (pepper). Numerous segmental duplicates were found in the moso bamboo *LBD* gene family. Gene expression profiles in four tissues showed that the *LBD* genes had different spatial expression patterns. qRT–PCR assays with the Short Time-series Expression Miner (STEM) temporal expression analysis demonstrated that six genes (*PeLBD20*, *PeLBD29*, *PeLBD46*, *PeLBD10*, *PeLBD38*, and *PeLBD06*) were consistently up-regulated during the rapid growth and development of bamboo shoots. In addition, 248 candidate target genes that function in a variety of pathways were identified based on consensus LBD binding motifs.

**Conclusions:**

In the current study, we identified 55 members of the moso bamboo transcription factor LBD and characterized for the first time. Based on the short-time sequence expression software and RNA-seq data, the PeLBD gene expression was analyzed. We also investigated the functional annotation of all *PeLBDs*, including PPI network, GO, and KEGG enrichment based on String database. These results provide a theoretical basis and candidate genes for studying the molecular breeding mechanism of rapid growth of moso bamboo.

**Supplementary Information:**

The online version contains supplementary material available at 10.1186/s12870-021-03078-3.

## Background

Moso bamboo (*Phyllostachys edulis*) is a non-timber forestry species from the subfamily Bambusoideae (Poaceae) that is native to Asia [1]. It has a wide distribution, a high economic value, and a broad range of industrial uses, and it plays an important role in soil and water conservation and climate regulation because of its propensity for fast growth [2]. The ability of moso bamboo shoots to undergo “burst growth” has gradually gained the attention of researchers and motivated investigations into the molecular mechanisms that underlie this rapid growth [3, 4]. However, the molecular mechanisms underlying the rapid growth of moso bamboo shoots are still unclear.

Transcription factors (TFs) regulation controls many important plant developmental processes such as cell morphogenesis, signal transduction, and environmental stress response by affecting gene expression [5]. Among them, the Lateral Organ Boundaries Domain (*LBD*) gene family, also known as *AS2/LOB*, is a class of TFs found only in higher plants. It was first identified through the insertion of enhancer traps and is expressed at the base of the proximal axis of the primary lateral organ of Arabidopsis [6, 7]. LBD TFs contain three specific conserved structural domains arranged from the N to the C terminus: the zinc finger-like C-block (C-block), the Gly-Ala-Ser-block (GAS-block), and the leucine-like zipper module (LX6LX3LX6L). The C-block contains four highly conserved cysteine motifs (CX2CX6CX3C), which are necessary for binding DNA. The GAS-block is located in the middle of the LOB structural domain and contains an invariant glycine residue. The leucine-like zipper module, consisting of about 30 amino acids at the C terminus, is involved in protein dimerization [8]. Based on the characteristics of the LOB structural domain, previous studies have divided the *LBD* gene family into two classes: class I and class II. Class I proteins contain both the conserved CX2CX6CX3C zinc finger-like motif and the leucine zipper module. By contrast, class II proteins contain only the conserved zinc finger-like structural domain [6, 7, 9]. However, recent studies have further subdivided class I into five subclasses, Ia–Ie, and class II into two subclasses, IIa and IIb [10, 11]. In addition, a variable C-terminal region occurs immediately after the leucine-like zipper module of the conserved LOB structural domain. This region can regulate the expression of downstream genes and is associated with nuclear targeting [12]. The LOB structural domain and the variable C terminus together form the expression structure of *LBD* genes [13].

Studies have shown that the LOB structural domain is involved not only in the regulation of early lateral organ development but also in additional processes such as tissue regeneration and responses to stress and pathogen infestation [14–16]. Expression of Arabidopsis *AtLOB*/*AtASL4* was first observed specifically at the base of the proximal axis of the lateral organ [6]. Iwakawa et al. found that *AtLBD6* (*AtAS2*) inhibited cell proliferation in the axial region through regulation of the *KNOX* gene, causing the leaf proximal-distal axis to develop symmetrically, forming spreading leaves. It also forms a negative feedback loop with AS1 and JAG to regulate lateral organ inflorescence development [7, 14, 15]. TFs *ARF7* and *ARF19* are expressed by cellular dedifferentiation process and act downstream of *LBD16* and *LBD18* to participate in the formation of Arabidopsis lateral roots [17]. *AtLBD15* regulates *WUS* expression and is involved in apical meristem cell differentiation [18]. *AtLBD38*, *AtLBD39*, and *AtLBD40* inhibited anthocyanin biosynthesis and also affected the nitrogen response [19], and *OsLBD37*, a homolog of this gene in rice, is also involved in nitrogen metabolism [20]. *AtLBD20* was the first gene identified to regulate the jasmonic acid (JA) signaling pathway, which plays a part in the response to plant pathogen *Fusarium oxysporum* [21]. Recent studies have shown that *STLBD2–6* is consistently highly expressed in potato stems under drought stress, suggesting that this gene may be associated with stem protection during drought [11].

The recent release of moso bamboo draft genome and chromosome level reference genome is the great potential for enabling genetic manipulation of the bamboo gene family [22, 23]. Based on the rapid development of genome sequencing technology, the identification of LBD TF genes has been reported in plants such as *Oryza sativa* [13], *Brassica napus* [24], *Gossypium raimondii* [25], *Vitis vinifera* [26], *Solanum tuberosum* [11], maize [27], and *Physcomitrella patens* [28]. The specific molecular functions of the LBDs have been validated in the model plants Arabidopsis and rice. LBD TFs have, however, not yet been characterized in moso bamboo. Thus, our present work aimed to identify all LBD family members of moso bamboo from the latest genomic database and to provide a comprehensive analysis of the protein characteristics, evolutionary relationships, conserved structures, repeat patterns, tissue specificity, and shoot rapid growth expression trends of *PeLBDs*. Our findings provide a theoretical basis for future studies on the functions of moso bamboo LBD genes and reveal their molecular mechanisms in rapid shoot growth.

## Results

### Identification of *LBD* genes in Moso bamboo

Fifty-nine putative *LBD* candidate genes were obtained from an HMMER3 search of the bamboo protein database using the plant LBD-type LOB model (Pfam PF03195) with an *E*-value threshold of ≤10^− 20^. We removed redundant genes and verified the presence of conserved domains and motifs to arrive at a final set of 55 *LBD* family members (Table 1). The genes were renamed *PeLBD01*–*PeLBD55* based on their positions on the chromosomal scaffolds.

**Table 1:** Detailed information on 55 PeLBD genes of moso bamboo and their encoded proteins

Gene Name	Gene ID	Type	Chromosome location	Size (aa)	MW (kDa)	pI	Stability	A.I.	GRAVY	Predicted Location
*PeLBD01*	PH02Gene35317.t1	Id	S3:569260–570,698	251	28.00	5.97	U	55.7	−0.537	Nucleus
*PeLBD02*	PH02Gene25030.t1	IIb	S4:11477296–11,478,570	224	23.10	8.10	U	78.88	0.115	Nucleus
*PeLBD03*	PH02Gene22099.t1	Id	S5:2535208–2,537,046	210	23.04	6.89	U	71.62	−0.346	Nucleus
*PeLBD04*	PH02Gene50302.t1	Ia	S6:43558391–43,560,298	229	24.37	8.45	U	73.14	−0.208	Nucleus
*PeLBD05*	PH02Gene44094.t1	Ia	S7:12271927–12,273,185	190	20.66	6.21	U	80.11	−0.125	Nucleus
*PeLBD06*	PH02Gene41745.t1	Ib	S9:2972347–2,973,626	211	22.34	6.13	U	82.51	0.056	Nucleus
*PeLBD07*	PH02Gene29604.t2	Ia	S9:7632319–7,634,241	237	24.61	8.83	U	79.66	−0.002	Nucleus
*PeLBD08*	PH02Gene42286.t1	Ia	S9:28982937–28,983,619	192	20.83	6.21	U	76.77	−0.174	Nucleus
*PeLBD09*	PH02Gene50136.t1	Ia	S9:40064587–40,068,578	265	27.26	8.22	S	66.75	−0.106	Nucleus
*PeLBD10*	PH02Gene30227.t1	IIa	S9:65163124–65,165,411	306	32.75	6.01	U	71.57	−0.42	Nucleus
*PeLBD11*	PH02Gene13066.t1	IIb	S10:8461781–8,463,035	267	28.41	7.53	U	81.87	0.02	Nucleus
*PeLBD12*	PH02Gene18918.t1	Ia	S11:875697–876,960	192	20.96	7.03	U	72.81	−0.31	Nucleus
*PeLBD13*	PH02Gene49654.t1	Ia	S12:880379–881,049	95	10.26	9.07	U	63.79	−0.08	Nucleus
*PeLBD14*	PH02Gene39836.t1	Ie	S12:50000380–50,001,379	236	26.40	5.49	U	70.81	−0.556	Nucleus
*PeLBD15*	PH02Gene47164.t1	Ia	S13:938790–939,778	187	20.64	7.65	U	77.33	−0.222	Nucleus
*PeLBD16*	PH02Gene23308.t1	Id	S13:43857925–43,859,085	226	24.93	5.33	U	57.04	−0.371	Nucleus
*PeLBD17*	PH02Gene15982.t1	Ic	S13:51796126–51,796,722	132	14.55	8.56	U	79.32	−0.152	Nucleus
*PeLBD18*	PH02Gene44004.t1	Ie	S13:97805306–97,807,157	347	37.32	4.86	U	71.53	−0.469	Nucleus
*PeLBD19*	PH02Gene32403.t1	Id	S13:103162261–103,165,624	211	22.94	7.08	U	77.35	−0.303	Nucleus
*PeLBD20*	PH02Gene22644.t1	IIa	S14:24538910–24,542,091	493	52.67	7.86	U	77.32	−0.363	Nucleus
*PeLBD21*	PH02Gene22183.t1	Ie	S14:33873927–33,875,581	194	21.21	6.34	U	58.61	−0.445	Nucleus
*PeLBD22*	PH02Gene12829.t1	Id	S14:60975289–60,976,389	220	23.52	8.20	U	83	−0.091	Nucleus
*PeLBD23*	PH02Gene03666.t1	Ia	S14:67423344–67,424,785	243	25.49	5.70	U	79.3	−0.077	Nucleus
*PeLBD24*	PH02Gene07923.t1	Ia	S14:76595025–76,598,346	255	26.45	8.21	S	73.96	−0.056	Nucleus
*PeLBD25*	PH02Gene06893.t1	Ie	S14:87968952–87,970,940	226	24.08	9.44	U	70.13	−0.326	Nucleus
*PeLBD26*	PH02Gene49769.t1	Ia	S14:99090637–99,093,273	244	25.26	8.47	U	73.69	−0.045	Nucleus
*PeLBD27*	PH02Gene20213.t1	IIa	S14:103753377–103,754,627	291	31.57	6.50	U	83.81	−0.298	Nucleus
*PeLBD28*	PH02Gene10461.t1	IIb	S15:14408150–14,410,891	223	23.13	7.55	U	78.03	−0.062	Nucleus
*PeLBD29*	PH02Gene25718.t1	IIb	S15:26506527–26,507,870	202	20.94	8.81	U	80.3	0.039	Nucleus
*PeLBD30*	PH02Gene00094.t1	Ib	S15:52345901–52,347,007	210	22.67	6.28	U	76.81	−0.285	Nucleus
*PeLBD31*	PH02Gene14134.t1	Id	S15:57752542–57,754,961	245	25.43	8.25	U	75.84	−0.189	Nucleus
*PeLBD32*	PH02Gene03608.t1	Id	S15:73887001–73,888,432	235	24.82	5.58	U	65.45	−0.14	Nucleus
*PeLBD33*	PH02Gene03609.t1	Id	S15:73891484–73,892,368	176	18.60	5.88	U	62.39	−0.169	Nucleus
*PeLBD34*	PH02Gene19411.t1	Ia	S15:77109714–77,112,202	190	20.39	8.85	U	71.42	−0.275	Nucleus
*PeLBD35*	PH02Gene10736.t1	Ia	S16:12222747–12,226,587	253	26.33	7.61	S	78.02	−0.036	Chloroplast, Nucleus
*PeLBD36*	PH02Gene17288.t1	Ia	S16:22162402–22,163,857	240	25.23	5.91	U	77.04	−0.115	Nucleus
*PeLBD37*	PH02Gene00718.t1	Id	S16:30110878–30,114,440	277	29.62	8.94	U	92.67	0.017	Nucleus
*PeLBD38*	PH02Gene37745.t1	IIa	S16:78665848–78,666,786	193	20.80	4.96	U	87.46	−0.161	Nucleus
*PeLBD39*	PH02Gene15546.t1	Ia	S16:114628136–114,630,170	245	25.29	6.94	U	78.16	0.016	Nucleus
*PeLBD40*	PH02Gene04162.t1	IIa	S16:118919168–118,920,519	281	30.30	6.18	U	82.31	−0.272	Nucleus
*PeLBD41*	PH02Gene16270.t1	Id	S17:1406541–1,409,381	256	28.21	5.97	U	88.05	−0.155	Nucleus
*PeLBD42*	PH02Gene04387.t1	Ib	S18:2408520–2,410,099	208	22.07	5.83	U	88.37	0.082	Nucleus
*PeLBD43*	PH02Gene40936.t1	Ia	S18:7045761–7,047,688	238	24.80	8.83	U	77.73	−0.01	Nucleus
*PeLBD44*	PH02Gene44534.t1	Ib	S19:29729602–29,731,436	258	28.10	8.26	U	75.74	−0.214	Nucleus
*PeLBD45*	PH02Gene06695.t1	Ic	S21:9838964–9,839,798	150	16.56	9.32	U	63.33	−0.527	Nucleus
*PeLBD46*	PH02Gene28345.t1	Ia	S21:31837259–31,840,382	193	20.54	9.04	U	75.39	−0.236	Nucleus
*PeLBD47*	PH02Gene26300.t1	Ia	S21:39362544–39,363,368	234	24.88	6.65	U	74.02	−0.246	Nucleus
*PeLBD48*	PH02Gene36754.t2	IIb	S21:39969712–39,971,881	221	22.92	8.10	U	85.34	0.037	Nucleus
*PeLBD49*	PH02Gene27319.t1	IIb	S21:50445998–50,447,235	200	20.62	8.42	U	81.55	0.027	Nucleus
*PeLBD50*	PH02Gene18619.t1	Ib	S21:83920941–83,922,562	212	22.59	6.18	U	81.13	−0.157	Nucleus
*PeLBD51*	PH02Gene11011.t1	Id	S21:107271840–107,272,932	270	28.75	6.03	U	69.22	−0.264	Nucleus
*PeLBD52*	PH02Gene11012.t1	Id	S21:107279627–107,280,827	176	18.50	5.64	U	62.39	−0.172	Nucleus
*PeLBD53*	PH02Gene43128.t1	Id	S22:638099–639,560	215	23.89	6.11	U	60.42	−0.385	Nucleus
*PeLBD54*	PH02Gene07094.t1	Ic	S22:6994313–6,994,963	167	18.43	6.36	U	70.3	−0.422	Nucleus
*PeLBD55*	PH02Gene26797.t1	Ie	S22:25560136–25,561,251	315	35.19	4.78	U	69.14	−0.685	Nucleus

*MW* molecular weight, *pI* isoelectric point, *A.I* aliphatic index, *GRAVY* grand average of hydropathicity score

Proteins encoded by the 55 *LBD* genes contained 95 (*PeLBD13*) to 493 (*PeLBD20*) amino acids, and their molecular weights (MWs) ranged from 10.25 (*PeLBD13*) to 52.67 kDa (*PeLBD20*). Approximately 80% of the LBD proteins had MWs of 20–30 kDa. Their predicted isoelectric point (pI) ranged from 4.78 (*PeLBD55*) to 9.44 (*PeLBD25*). Instability index calculations predicted that 51 (95%) of the LBD proteins were unstable in vitro*. PeLBD09*, *PeLBD24*, and *PeLBD35* had instability indices less than 40 and were classified as stable proteins. Aliphatic amino acid indices showed that the thermal stability of the proteins ranged from 55.701 to 92.67, indicating that differences in their thermal stability were relatively minor. The grand average of hydropathicity (GRAVY) score was negative for 47 (86%) of the LBD proteins, demonstrating that they were predominantly hydrophilic. Cell-PLoc subcellular localization predictions suggested that almost all LBD proteins were located in the nucleus (Table 1).

### Phylogenetic analysis and conserved sequence alignment

**Fig. 1:**
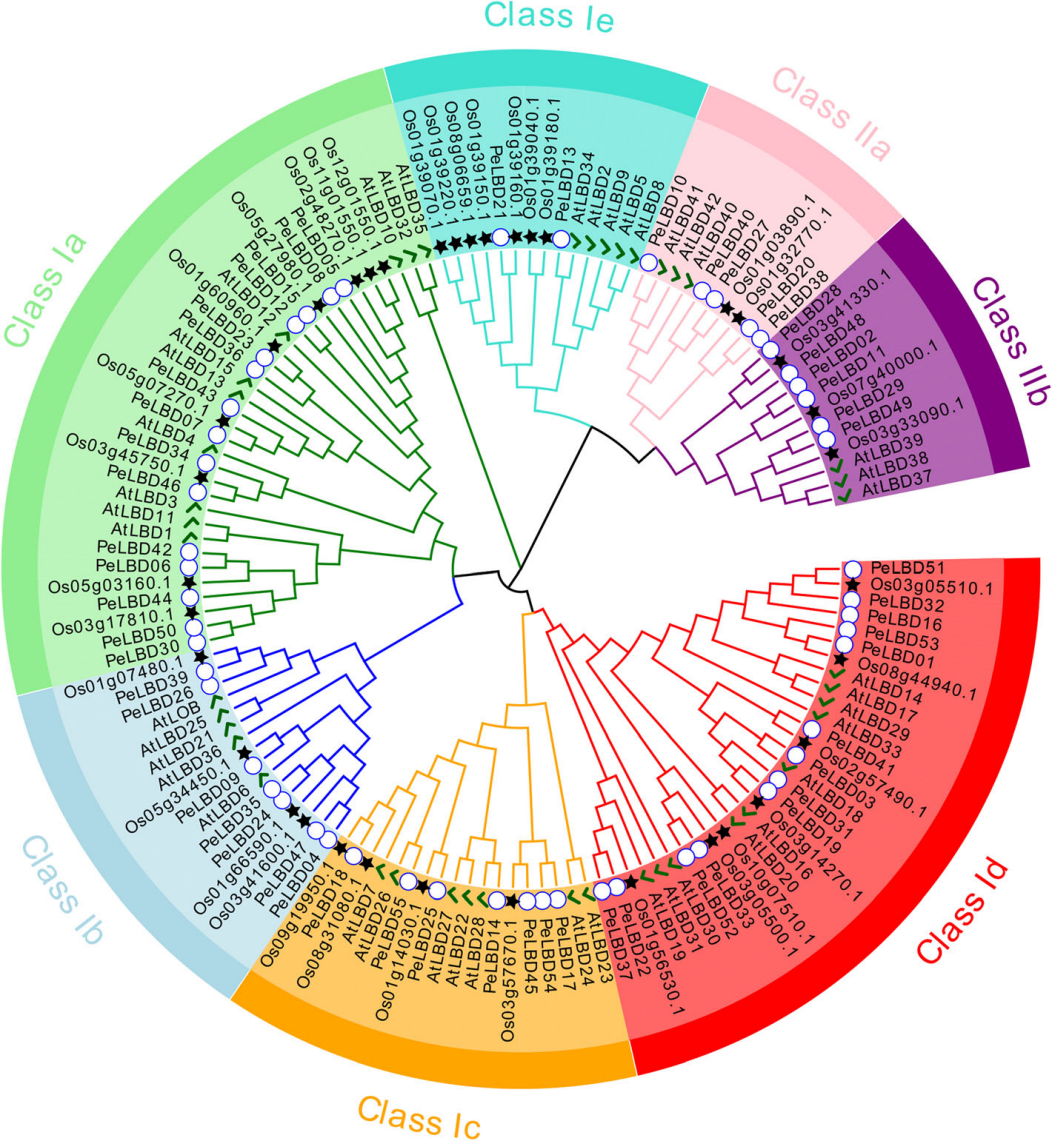
Phylogenetic analysis of full-length LBD protein sequences from *Phyllostachys edulis* (Pe, moso bamboo), *Arabidopsis thaliana* (At, Arabidopsis), and *Oryza sativa* (Os, rice). MUSCLE was used to build a multiple sequence alignment, and MEGA7.0 was used to construct a maximum likelihood (ML) phylogenetic tree with 1000 bootstrap replicates. White circles, green check marks, and black stars indicate bamboo, Arabidopsis, and rice sequences, respectively

To clarify the evolutionary relationships between moso bamboo PeLBD proteins and LBD proteins of other species, an Maximum Likelihood (ML) phylogenetic tree was constructed using the amino acid sequences of 55 moso bamboo LBDs, 36 rice LBDs, and 43 Arabidopsis LBDs (Fig. 1). Based on well-established Arabidopsis and rice LBD family classifications [13], the LBD proteins were classified into two major groups, class I and class II. Class I had 112 members: 29, 44, and 39 in rice (25.9%), moso bamboo (39.3%), and Arabidopsis (34.8%), respectively. Class II had 24 members: 6, 11, and 7 in rice (25%), moso bamboo (44%), and Arabidopsis (28%), respectively. Class I could be subdivided into five subclasses (Ia–Ie), and class II could be subdivided into IIa and IIb. Subclass Ia had the most members (34), and subclass IIa had the fewest (10). Each species contained members of each subclass, indicating that all seven subclasses were present among both monocots and dicots. Phylogenetic relationships indicated that the LBD proteins of moso bamboo showed greater homology to those of rice than to those of Arabidopsis.

The number of *LBD* genes in moso bamboo (55) was similar to that in maize (44), *S. tuberosum* (43), and Arabidopsis (43) but significantly different from that in *B. napus* (126), and mosses (31). Compared to class II, class I had more members in the different species (Supplemental Fig. 1).

**Fig. 2:**
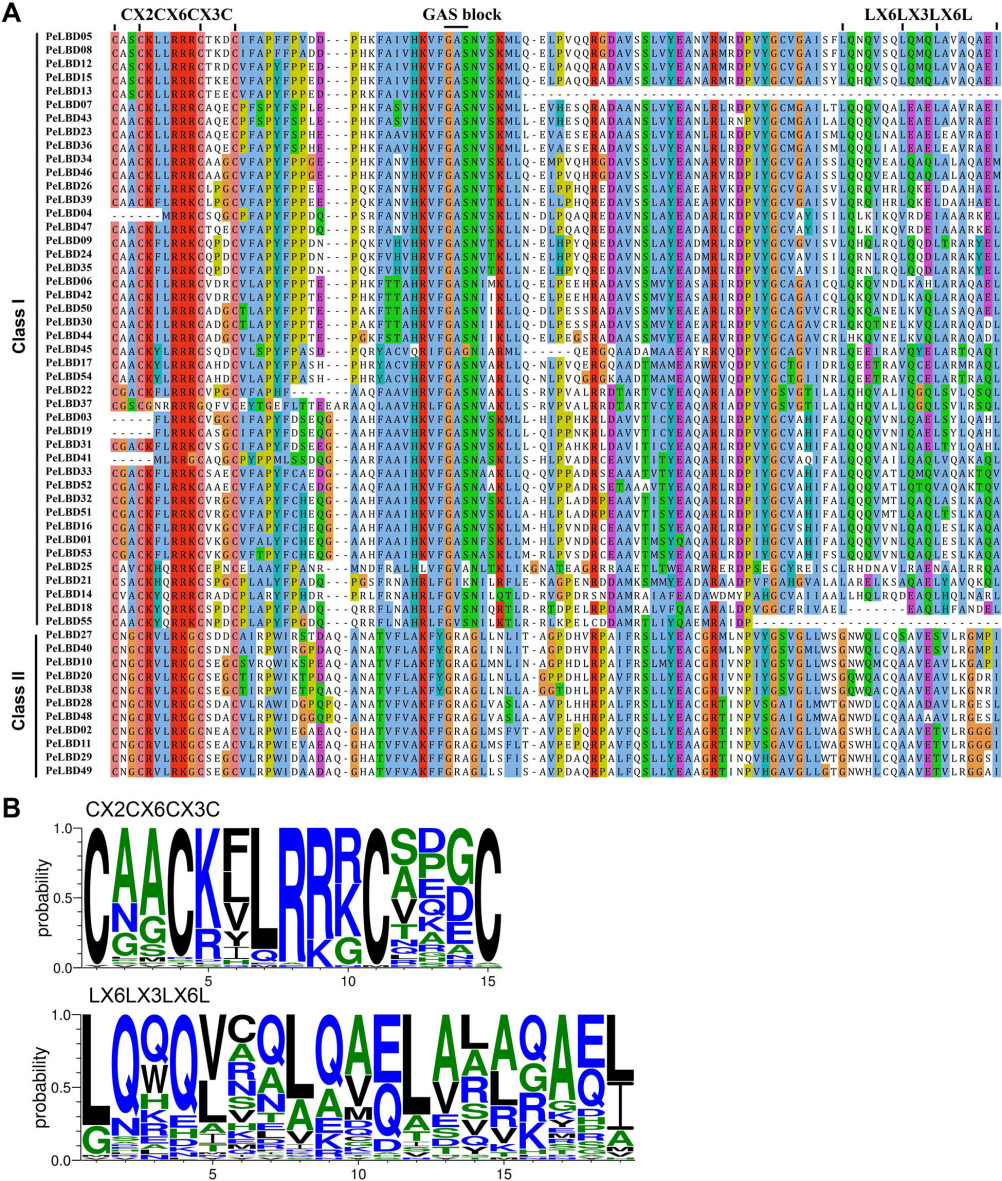
Conserved domains of the moso bamboo PeLBD proteins. **a** The CX2CX6CX3C zinc finger-like domain was present in all 55 predicted PeLBD protein sequences, whereas the leucine zipper-like motif (LX6LX3LX6L) was present only in the class I PeLBD proteins. **b** Sequence alignment of two protein domains by MUSCLE and conserved motif logos generated by the WebLogo program

Multiple sequence alignments were created for the 55 PeLBD proteins to investigate the presence and locations of conserved protein domains. All LBD family members contained a highly conserved LOB region at the N terminus, which consisted of approximately 100 amino acids (Fig. 2a). Multiple sequence comparisons showed that all LBD proteins contained the zinc finger-like structural domain (Fig. 2b). By contrast, the leucine zipper-like structural domain was only present in class I PeLBD proteins, similar to results from other plant species.

### Gene structure and motif composition analysis

**Fig. 3:**
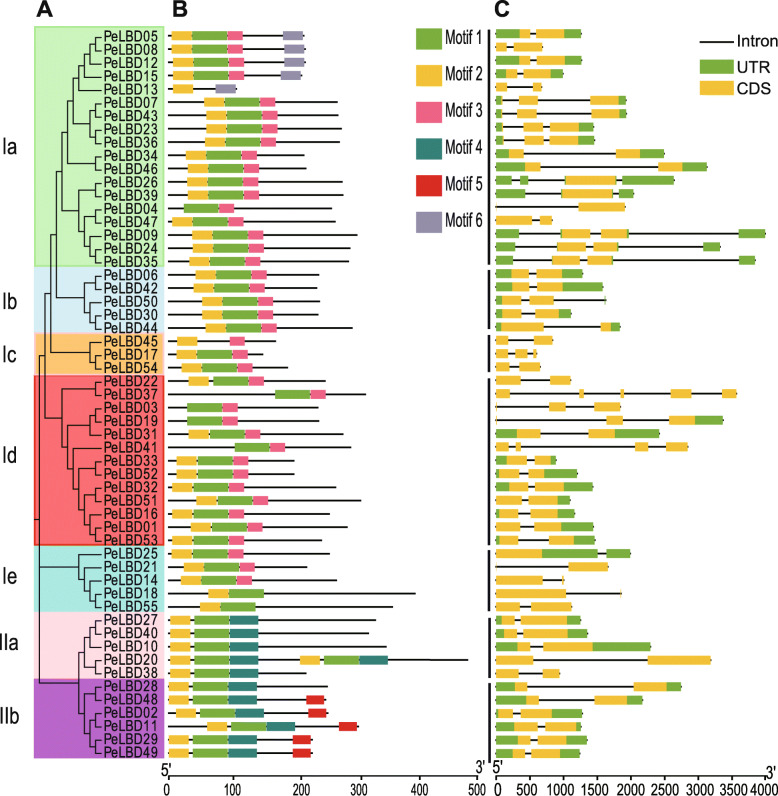
The phylogenetic relationships, conserved motifs, and gene structures of PeLBD proteins and *PeLBD* genes. **a** An Maximum Likelihood (ML) phylogenetic tree of the moso bamboo proteins was constructed from full-length sequences in MEGA 7.0 with 1000 bootstrap replicates. **b** Distribution of conserved motifs in the PeLBD proteins. The colored boxes represent motifs 1–6. The scale bar indicates 100 aa. **c** The gene structures of the *PeLBD* genes, including introns (black lines), exons (yellow rectangles), and untranslated region (UTRs, green rectangles). The scale bar indicates 0.5 kb

To further investigate the evolutionary relationships among the moso bamboo PeLBDs, we constructed a second phylogenetic tree using only the full-length PeLBD protein sequences. This analysis confirmed that class I was divided into five subclasses, Ia, Ib, Ic, Id, and Ie, with 18, 5, 3, 13, and 5 members, respectively. Class II was divided into two subclasses IIa and IIb, which had 5 and 6 members (Fig. 3a). We identified six highly conserved motifs in each LBD protein using MEME (Fig. 3b and Supplemental Fig. 2). Almost all LBDs contained motif 1 and motif 2, and these constituted the most highly conserved part of the LOB domain. The relative positions of motifs were similar in most sequences, with the exception of *PeLBD20* in subclass IIa, which had an extra set of motifs 1, 2, and 4. Interestingly, some motifs were detected only in specific subfamilies. For example, motif 6 and motif 5 were only found in subclasses Ia and IIb, respectively.

The number of introns in each moso bamboo *LBD* gene ranged from one to four (Fig. 3c). About 70% of the genes contained one intron, nine genes contained two introns, five genes contained three introns, and one gene contained four introns (*PeLBD37*). The structures of *LBD* genes on the same phylogenetic branch were generally similar.

### Analysis of *cis*-elements in *PeLBD* promoters

**Fig. 4:**
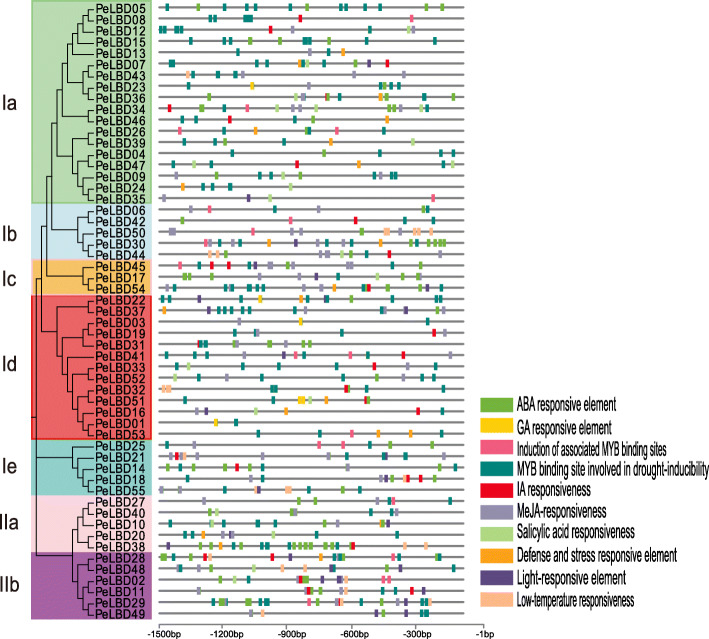
Diagram of *cis*-acting elements identified in the 1500-bp upstream promoter regions of the moso bamboo *PeLBD* genes. Rectangular boxes of different colors represent individual *cis*-acting elements, and some elements overlap with one another

The *cis*-acting elements are non-coding DNA sequences in gene promoters that regulate the transcription of their associated genes. We identified ten important *cis*-acting elements 1500 bp upstream of the moso bamboo *PeLBD* genes using PlantCARE software (Fig. 4). Numerous *cis*-elements in the *PeLBD* promoters were associated with the response to hormones: abscisic acid (ABRE), MeJA (CGTCA-motif), gibberellic acid (GARE-motif), salicylic acid (TCA-element), and auxin (TGA-element). Some promoters contained stress-related elements, particularly TC-rich repeats involved in defense and stress response and low temperature response (LTR)-related elements. Interestingly, all *LBD* promoters contained MYB binding site involved in the induction of drought, high salt, and low temperature responses. MYB was the most abundant element (> 200). In addition, the light-responsive element (LRE) was present in the promoters of 28 *PeLBD* genes (Fig. 4 and Supplemental Fig. 3). These results suggest that the expression of *LBD* genes in moso bamboo is regulated by *cis-*elements associated with plant developmental processes and abiotic stress tolerance.

### Chromosomal location and Synteny analysis

**Fig. 5:**
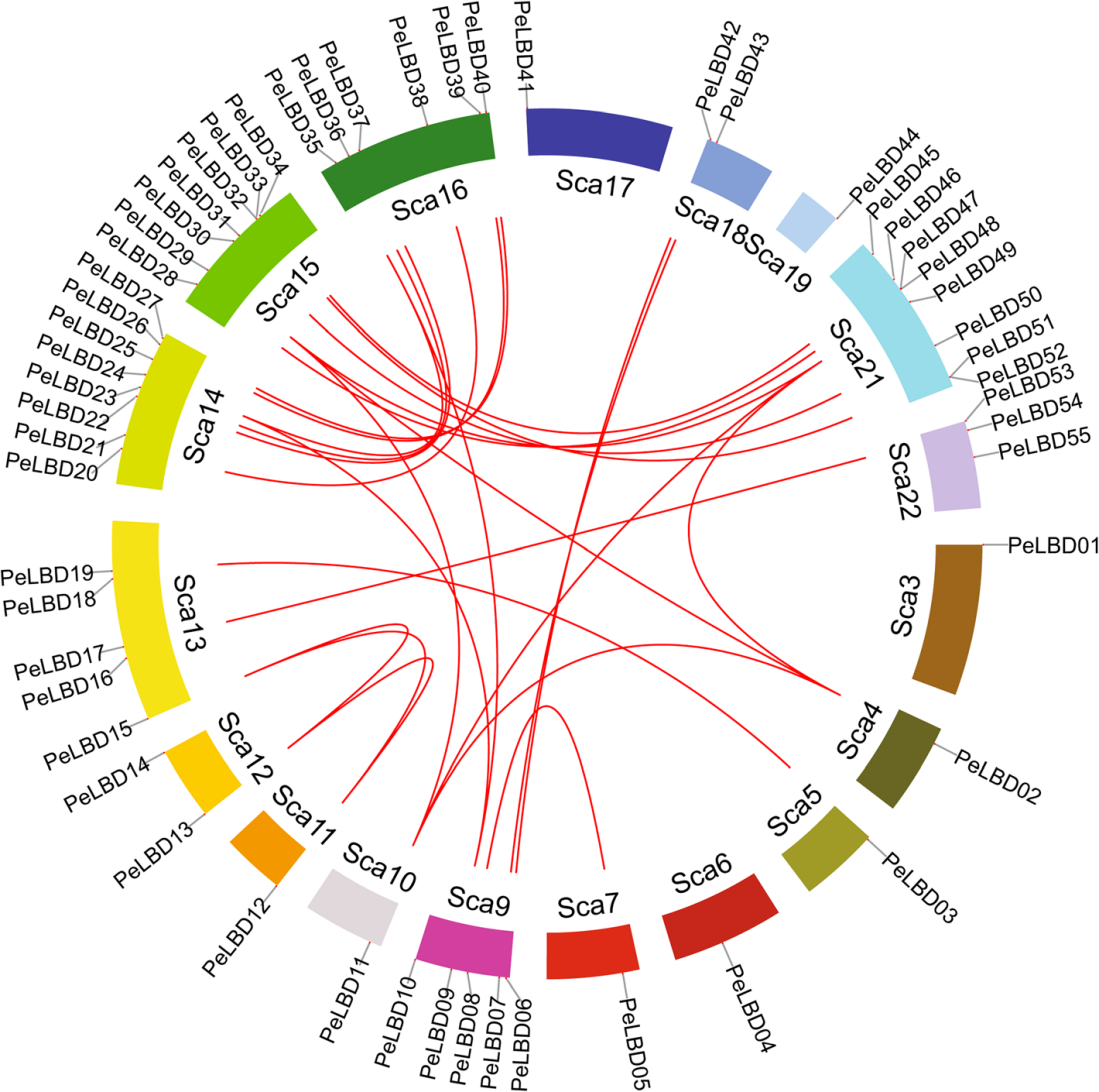
Chromosomal distribution and inter-chromosomal relationships of *PeLBD* genes. Red lines connect duplicate *PeLBD* gene pairs

The moso bamboo *PeLBD* genes were non-uniformly distributed across the 18 chromosome scaffolds of moso bamboo (Fig. 5). The largest number were found on scaffolds 14 and 21 (8, 14.54%), followed by scaffolds 15 (7, 12.72%), 16 (6, 10.90%), 9 and 13 (5, 9%), 22 (3, 5.45%), and 12 and 18 (2, 3.63%). All other chromosomes contained a single *PeLBD* gene. Small gene clusters were found on scaffolds 15 and 21, based on the definition of gene clusters.

Gene duplication events are prevalent in all species; they can give rise to new functional genes and drive species evolution [29]. We therefore used MCScanX genome synteny analysis to explore duplications within the moso bamboo *LBD* gene family (Fig. 5). Twenty-six gene pairs appeared to have arisen from segmental duplications, and 42 (76%) of the *PeLBD* genes were replicated and retained after whole genome duplications (WGDs).

### Evolutionary analysis of the *PeLBD* genes

**Fig. 6:**
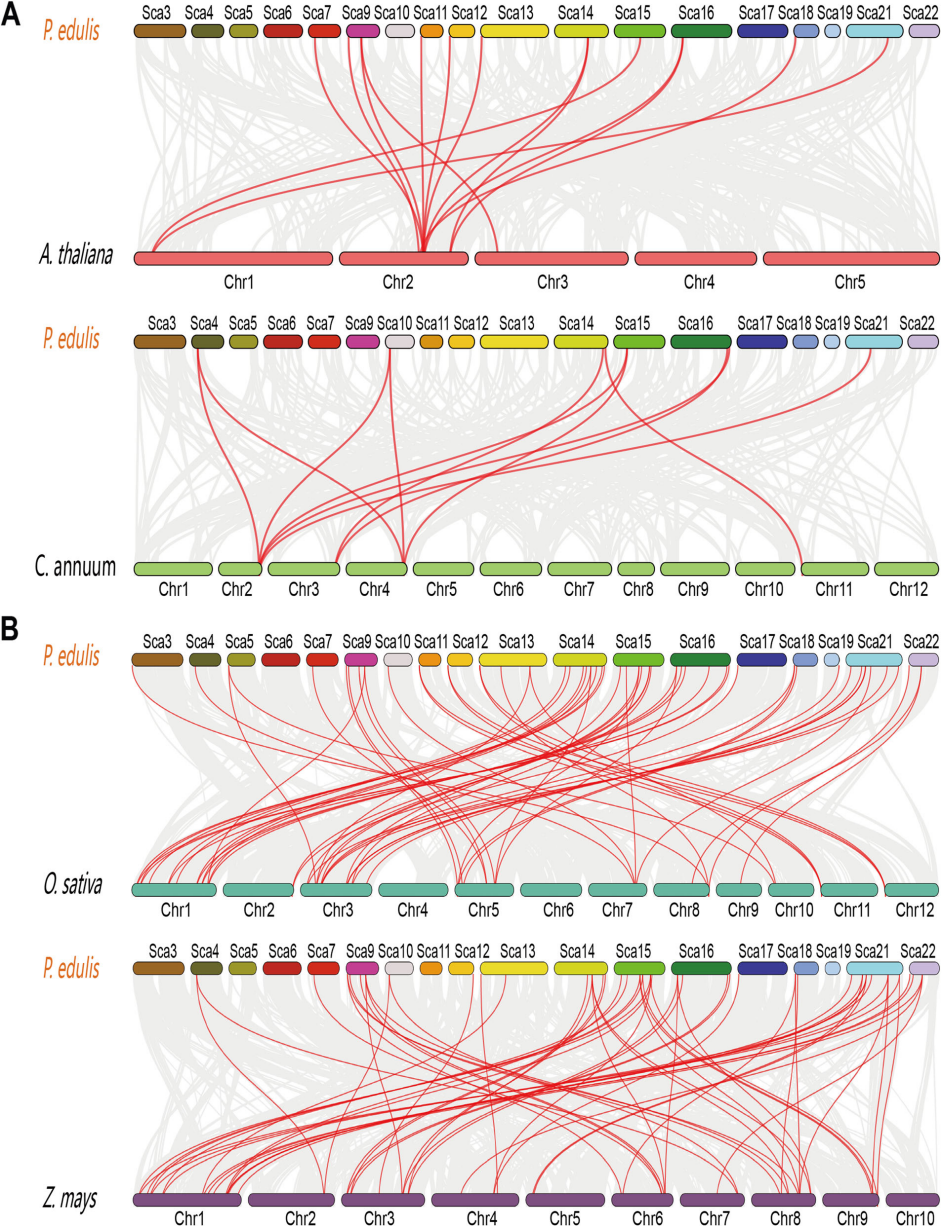
Synteny analysis of the moso bamboo genome with two monocot (**a**) and two dicot (**b**) plant genomes. The gray lines represent aligned blocks between the paired genomes, and the red lines indicate syntenic *LBD* gene pairs

To further investigate gene duplications in the *LBD* gene family, we performed genome-to-genome synteny analysis between moso bamboo and four representative plants: two dicots (Arabidopsis and pepper) (Fig. 6a) and two monocots (rice and maize) (Fig. 6b). Fourteen, 11, 58, and 60 moso bamboo *LBD* genes were syntenic with those of Arabidopsis, pepper, rice, and maize, respectively. There was, therefore, greater collinearity between the bamboo and monocot genomes than between the bamboo and dicot genomes. Furthermore, the rice *LBD* genes all had corresponding orthologs in moso bamboo, and most of them had more than two orthologs, suggesting that moso bamboo has undergone additional WGD event(s) during its evolution.

To investigate evolutionary constraints and selection pressures on the *PeLBD* genes, we calculated Ka, Ks, and Ka/Ks for 17 homologous *PeLBD* gene pairs (Supplemental Table 1). Ks represents the background base substitution rate, and Ks values can, therefore, be used to predict the timing of whole genome duplications (WGD)events. The Ks values of the *PeLBD* gene pairs ranged from 0.0718 to 0.2392, indicating that a large-scale *PeLBD* gene duplication event occurred as early as 18.40 million years ago (MYA) and as recently as 5.52 MYA. The Ka/Ks values of the gene pairs were all less than 1.0, and these genes may therefore have undergone strong purifying selection during evolution.

### Expression patterns of *PeLBD*s in different tissues

**Fig. 7:**
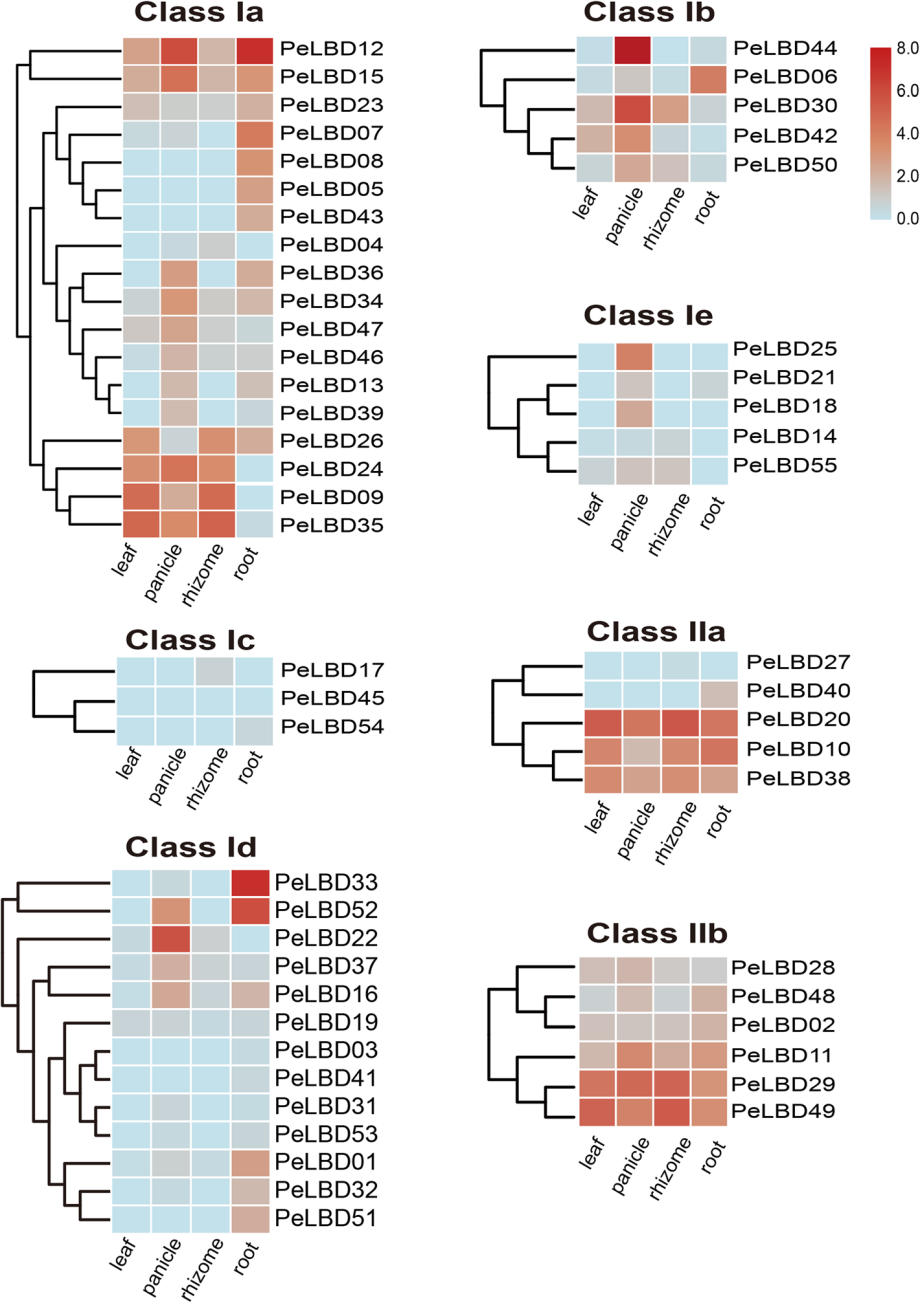
Expression heatmap of log2 (TPM + 1) values of 55 moso bamboo *PeLBD* genes in four different tissues: roots, rhizomes, panicles, and leaves. Based on the phylogenetic results (Fig. 2a), the *LBD* gene family was divided into seven subgroups: classes Ia–Ie and classes IIa and IIb. Red indicates high expression, and blue indicates low expression

We used published transcriptome data to explore the expression patterns of *PeLBD* genes in four different tissues: roots, rhizomes, panicles, and leaves. Gene expression was calculated as Transcripts Per Kilobase Million (TPM). The results showed that 55 *PeLBD* genes had significantly different expression patterns among tissues (Fig. 7). Fifteen *PeLBD* genes had detectable expression in all tissues, suggesting that they participate in the development or physiology of multiple tissues. Four genes (*PeLBD12*, *PeLBD44*, *PeLBD30*, and *PeLBD22*) were apparently highly expressed in panicles, suggesting that they may be involved in the development and function of bamboo flowers. Specially, *PeLBD12* and *PeLBD33* were highly abundant in roots (TPM > 100). Interestingly, subclass three Ic members were not expressed in any tissues, suggesting potential functional redundancy among subclass Ic *PeLBD*s. By contrast, six members of subclass IIb appeared to be expressed in all tissues, suggesting that they may have important functions in tissue growth. In summary, the results of transcriptome sequencing analysis confirm that *PeLBD* genes was significantly differentially expressed in a variety of tissues.

**Fig. 8:**
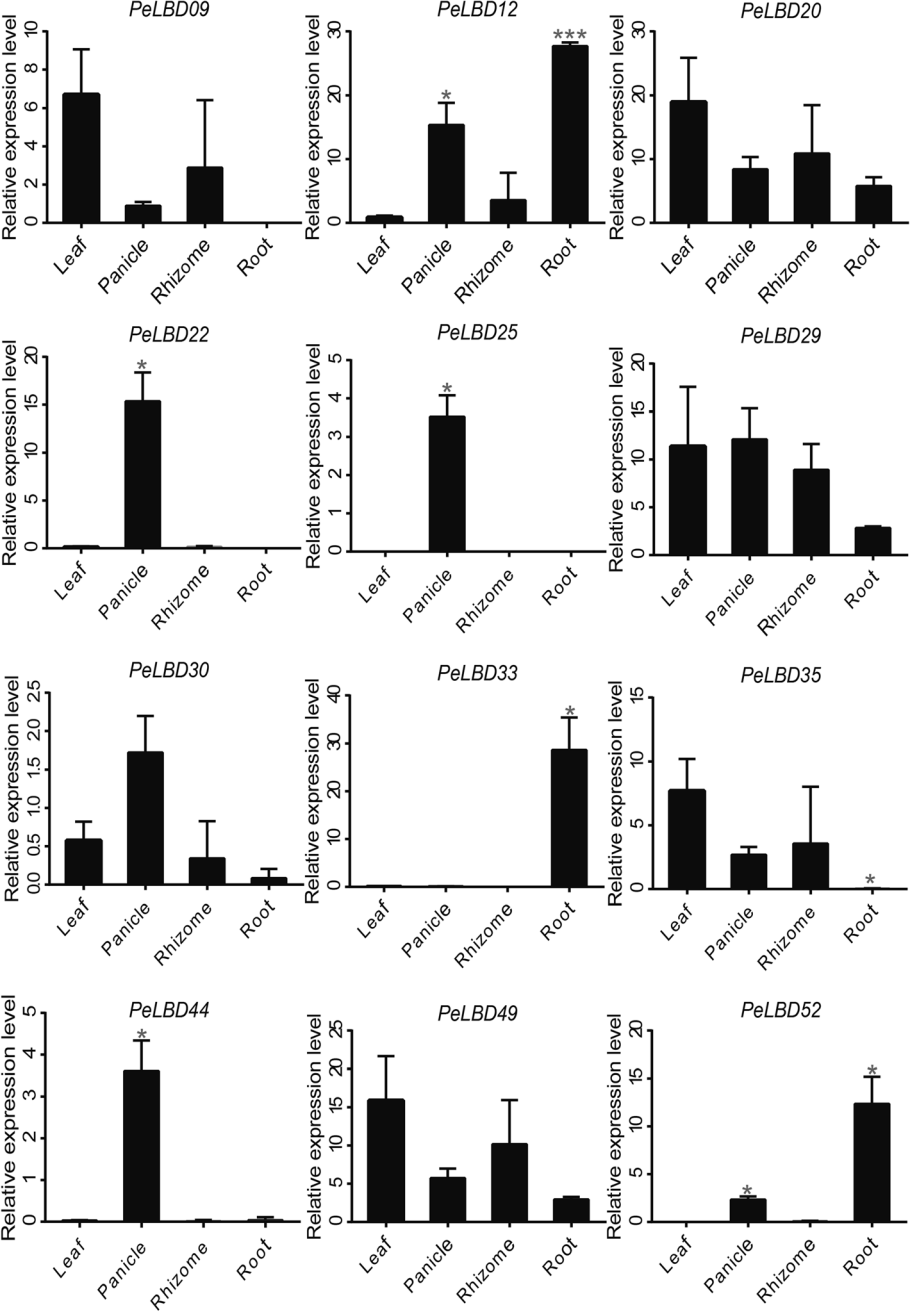
qRT–PCR analysis of 12 genes in four moso bamboo tissues (roots, rhizomes, panicles, and leaves). All experiments were performed independently at least three times. Error bars represent the standard deviation of three replicates. Asterisks indicate significant differences in transcript levels compared with those of leaf. (*P < 0.05, **P < 0.01, ***P < 0.001)

To verify the reliability of the transcriptome data, we also used qRT–PCR to further validate the expression of 12 *PeLBD* genes in four tissues: roots, rhizomes, panicles, and leaves (Fig. 8). Genes such as *PeLBD20* and *PeLBD29* were expressed in all organs, indicating that they may play a general role in the growth process. Among the same genes, LBDs were moderately expressed in the rhizomes compared to other tissues. However, some genes showed tissue-specific expression patterns. For example, *PeLBD22*, *PeLBD44*, *PeLBD25*, and *PeLBD29* were highly expressed in the panicles, and *PeLBD09*, *PeLBD20*, *PeLBD49*, and *PeLBD35* were highly expressed in the leaves. *PeLBD20* and *PeLBD49* had slightly lower expression in roots than in other tissues. Notably, *PeLBD33* was highly expressed only in the roots, suggesting that it may be a root-specific gene. Overall, the qRT–PCR results supported the results of the transcriptome sequencing analysis.

### Identification of genes associated with rapid development in bamboo shoots

**Fig. 9:**
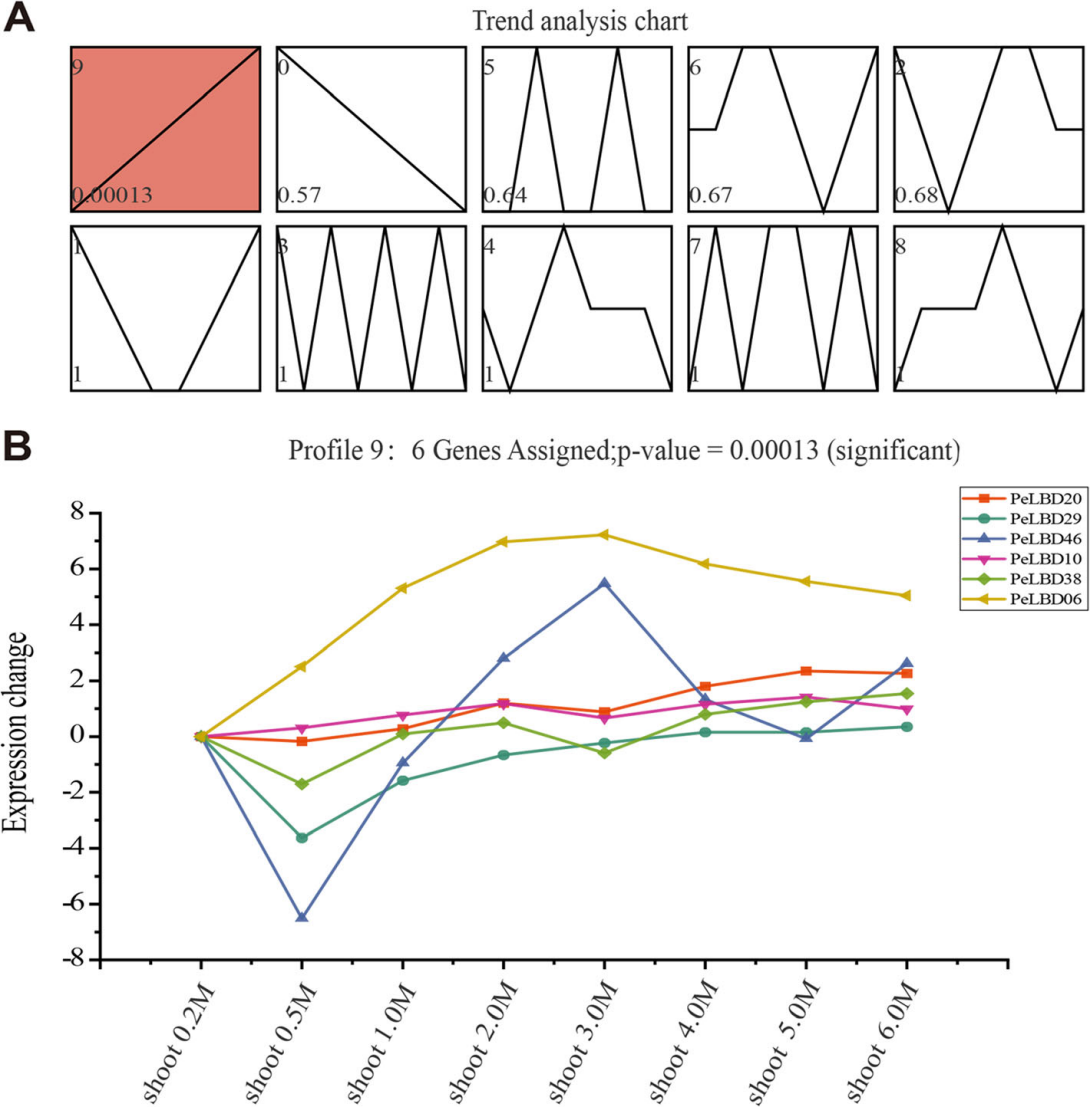
Time series expression analysis of moso bamboo *PeLBD* genes in shoots of different ages/growth heights. **a** Trend analysis graphs produced by the STEM algorithm. Each graph shows a gene expression change trend; the values in the upper and lower left corners indicate the number of genes assigned to the profile and the corresponding *P*-value. The line represents the trend in target gene expression over time. Only the red profile (profile 9) is significant. **b** Expression changes of the six genes in profile nine

To investigate the function of *PeLBD* genes in the rapid developmental pathway of shoots, we performed trend analysis of *LBD* gene expression profiles using the Short Time-series Expression Miner (STEM) software based on expression data from shoots of different ages/growth heights (0.2–6 m) during the fast growth period. A total of 10 expression trends were identified (Fig. 9a). The trend of significant enrichment (profile 9) showed a positive correlation with shoot development, suggesting that the genes in this profile were gradually upregulated during shoot growth. Six genes (*PeLBD20*, *PeLBD29*, *PeLBD46*, *PeLBD10*, *PeLBD38*, and *PeLBD06*) were assigned to profile 9, and their expression was generally upregulated during shoot development (i.e., it increased with increasing growth height) (Fig. 9b).

**Fig. 10:**
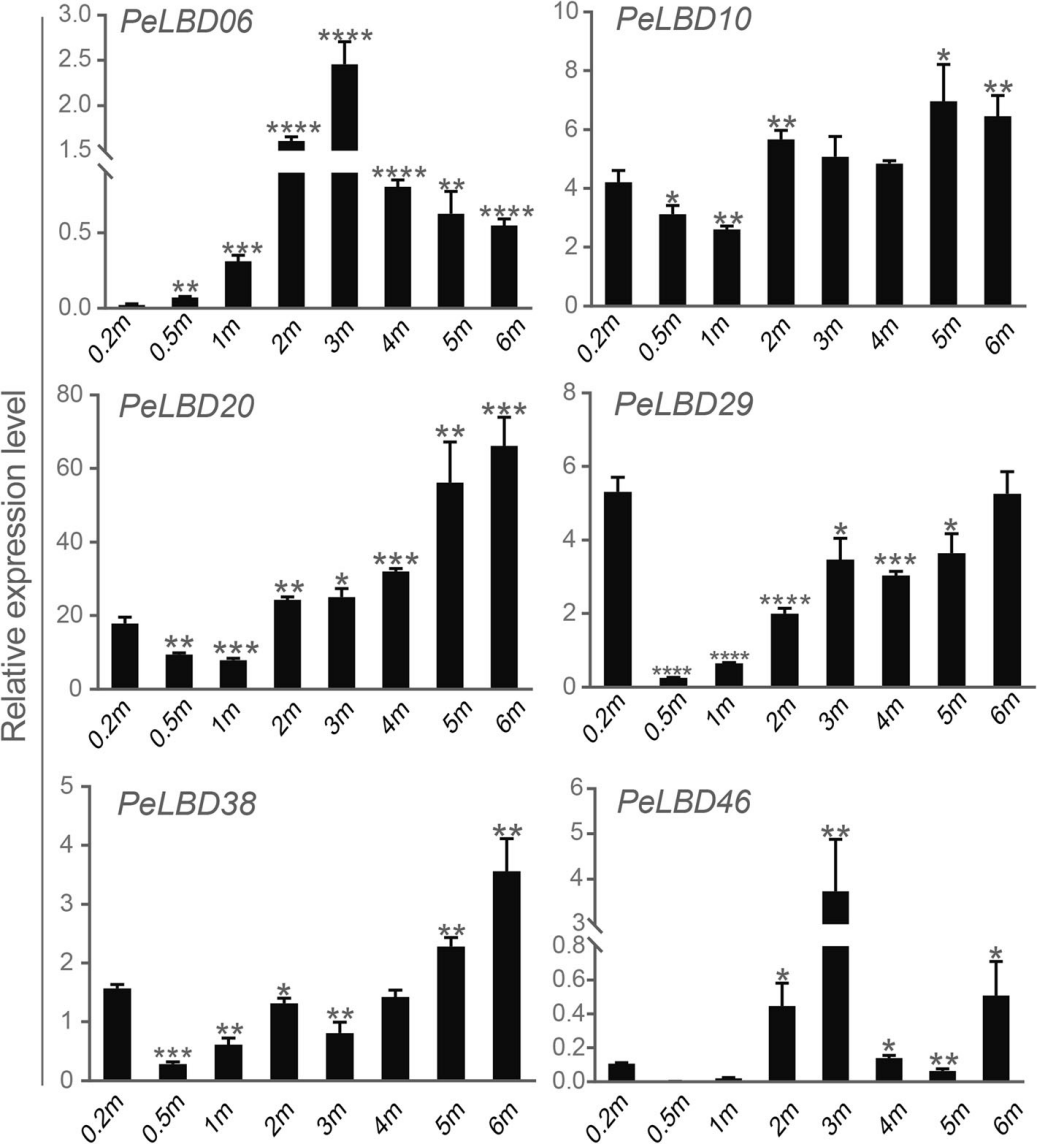
RT–qPCR analysis of six *PeLBD* genes whose expression was previously shown to increase in shoots of increasing height. All experiments were performed independently at least three times, and the data are expressed as the mean ± standard deviation (SD). Asterisks indicate significant differences in transcript levels compared with those of 0.2 m shoots. (*P < 0.05, **P < 0.01, ***P < 0.001, ****P < 0.0001)

To further validate the results of the STEM analysis, we further performed qRT–PCR for the six genes (see above) whose expression increased significantly during the rapid growth and development of bamboo shoots (Fig. 10). With the exception of *PeLBD46*, the genes all showed a general increase in expression with shoot developmental stage, although there were differences in the magnitude and timing of this increase. *PeLBD06* showed a significant increase in expression that peaked in 3 m shoots; *PeLBD29* showed the highest expression in 0.2 m shoots but then slowly rose again as shoots increased in height from 0.5 to 6 m. As lignification of bamboo shoots increased during later growth stages, the expression of *PeLBD20* and *PeLBD38* showed the strongest positive correlation with rapid shoot development, unlike the other four genes. Overall, the qRT–PCR results supported the results of the STEM temporal clustering analysis, which suggested that these *PeLBD* genes might play an important role in the rapid growth, development, and lignification of bamboo shoots.

### Construction of a PPI network and GO enrichment analysis

**Fig. 11:**
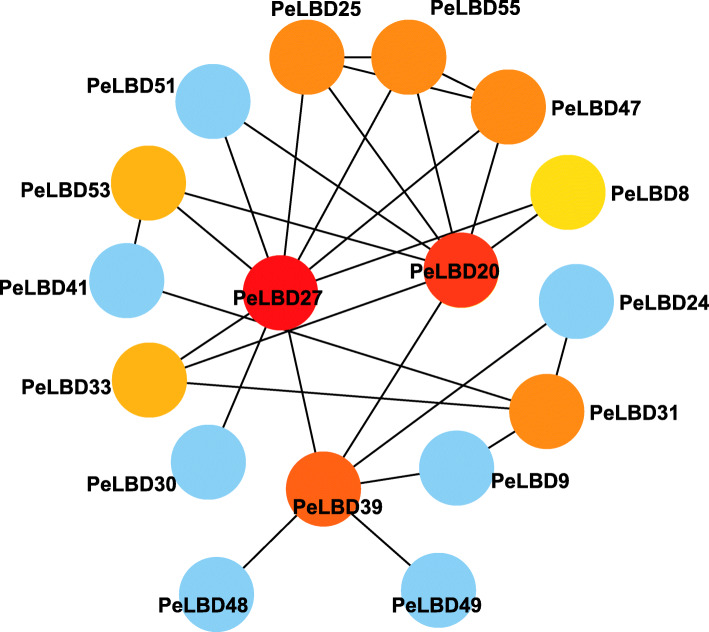
Protein–Protein Interaction (PPI) network of significant genes in moso bamboo. Nodes represent proteins, central nodes are indicated in red, and black lines indicate interactions between nodes. The darker the color, the more important the protein in the interaction network

We used the STRING database to predict potential interactions among the PeLBD proteins (Fig. 11). There were 17 nodes in the PeLBD protein interaction network, each of which interacted with other nodes. Some proteins exhibited direct interactions, such as *PeLBD49* and *PeLBD39*, whereas others exhibited more complex multigene interactions, such as *PeLBD25*, *PeLBD55*, and *PeLBD47*. Notably, *PeLBD20* and *PeLBD27* were predicted to be central nodes, radiating eight and nine connections to other genes, respectively.

**Fig. 12:**
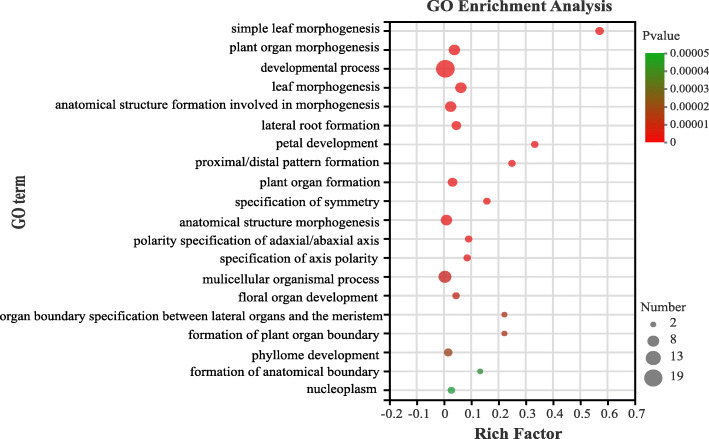
The top 20 enriched GO terms of the *PeLBD* genes. The horizontal axis indicates the enrichment factor, and the size of the circle indicates the number of genes annotated with a given GO term

To predict their biological functions, we performed GO annotation and enrichment analysis of the 55 PeLBD proteins. The top 20 GO terms are shown in Fig. 12. The strongest enrichment and the highest enrichment factor (0.58) were observed for the process of leaf morphogenesis, followed by the process of petal development (0.33). In addition, the largest number of genes (19) was associated with the GO term “developmental process.”

### Identification and annotation of PeLBD target genes

**Fig. 13:**
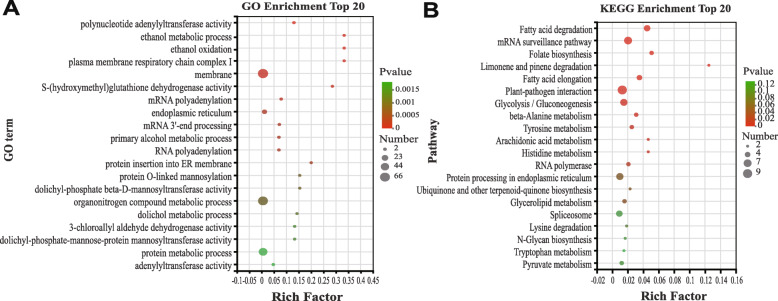
The top 20 GO terms (**a**) and KEGG pathways (**b**) enriched of candidate *PeLBD* target genes. The black circles indicate the number of target genes, and different colors indicate the *P*-value, ranging from 0 to 0.12

To identify the potential downstream target genes regulated by bamboo LBDs and determine their functions, consensus LBD motifs from the JASPAR database (Supplemental Fig. 4) were used to search the 2.0-kb promoter sequences upstream of the moso bamboo protein-coding genes. A total of 248 target genes were identified for further annotation and were classified into three major classes and 32 subclasses. Among the 248 genes, 89 received GO annotations, and 107 were mapped to the KEGG database. GO analysis showed that the terms cell part (GO:0044464), metabolic process (GO:0008152), and catalytic activity (GO:0003824) were assigned to many target genes (Supplemental Fig. 5). Among the top 20 GO terms enriched in the target gene set were ethanol metabolic process (GO:0006067), cell membrane fraction (GO:0016020), organonitrogen compound metabolic process (GO:1901564), and other significantly enriched terms (*P* < 0.05) (Fig. 13a). Likewise, in the KEGG analysis, the largest number of target genes (112) were assigned to the carbohydrate metabolism pathway (Supplemental Fig. 6). Among the top 20 KEGG pathways enriched in the target genes were fatty acid degradation (Ko00071), plant-pathogen interaction (Ko04326), folate biosynthesis (Ko00790), and mRNA surveillance pathway (Ko03015). These results suggest that PeLBDs can influence multiple pathways by regulating their target genes (Fig. 13b).

## Discussion

At present, the chromosome level reference genome of moso bamboo enables the comprehensive characterization of important gene families [22, 23]. Here, we identified 55 *PeLBD* genes from moso bamboo and divided them into two classes, class I (44, 80%) and class II (11, 20%) (Fig. 3a). These two classes were further divided into different subclasses. Previous studies found that 86% of the *LBD* genes in Arabidopsis and 88% of those in potato belonged to class I [11, 13] (Supplemental Fig. 1). Our results provide further evidence that the number of class I *LBD* members is substantially higher than that of class II members in different species. One hundred thirty-four *LBD* genes from several species were further classified into seven subclasses (Ia–Ie and IIa–IIb), and their phylogenetic relationships were generally consistent with those reported in previous studies [30, 31] (Fig. 1). Many homologous *PeLBD* gene pairs were expressed at similar levels (Fig. 7), suggesting that duplication of *LBD* genes in moso bamboo may have mainly led to functional redundancy.

Gene structure analysis revealed that some members within the same subclass have structural differences. For example, the number of introns in *PeLBD* genes from subclass Ia varies from 1 to 3. We speculate that members of subclass Ia may have undergone splicing or insertion of gene fragments during evolution [32, 33]. Nevertheless, the similar conserved sequences and gene structures within *LBD* subclasses suggest that genes within a subclass may generally have similar biological functions. Furthermore, comparisons of the LOB conserved structural domains showed that the complete leucine-like zipper motif was detected in all class I genes except *PeLBD13*, *PeLBD18*, and *PeLBD55*, suggesting that motifs in the LBD family are broadly conserved during evolution.

Multiple *cis-*acting elements located in gene promoters play a crucial role in signaling, and synergistic interactions among them can regulate complex biological processes. *AtLBD16* and *AtLBD29* have been reported to be involved in the growth hormone response and lateral root formation [34, 35]. *AtLBD20* (*AtASL21*) functions in plant disease resistance mediated by the jasmonate signaling pathway [21]. We found that the *LBD* promoters contained numerous motifs related to hormone regulation pathways, including those of abscisic acid, MeJA, and IAA. Thus, we conclude that *PeLBD* genes may also participate in the plant stress response. Interestingly, all *PeLBD* gene promoters have MYB elements that are involved in drought-inducibility, and MYB elements were the most common *cis*-elements detected (Supplemental Fig. 3). There are few previous studies on the response of *LBD* genes to abiotic stress, but recent work in potato has confirmed that the expression levels of *StLBD1–5* and *StLBD2–6* are down-regulated and up-regulated, respectively, under drought stress to maintain normal physiological functions [11]. Furthermore, members of the potato *LBD* family also contain numerous MYB elements. A part of MYB genes have been genetically transformed in Arabidopsis, wheat and rice, and it was confirmed that overexpression of MYB genes could improve the drought resistance of transgenic plants [36–38]. Overall, we suggest that bamboo *LBD*s may play an important role in the response to abiotic stresses, particularly drought. Nonetheless, the specific expression patterns of different *LBD* genes remain to be verified by further molecular biology experiments.

Gene duplication plays an important role in evolution by facilitating the generation of new genes and gene functions. There are three main evolutionary modes of gene duplication [39]: segmental duplication, tandem duplication, and translocation events. Segmental and tandem duplication most commonly underlie the expansion of plant gene families [39, 40]. Moso bamboo contains 55 *LBD* genes, 12 more than Arabidopsis, and has 1.27, 1.52, 1.25, 1.27, and 0.80 times as many *LBD* genes as Arabidopsis (43), rice (36), maize (44), potato (43), and *G. raimondii* (68), respectively. Although the genome size of moso bamboo (2051.7 Mb) is similar to that of its close relative, maize (2066.4 Mb), the number of *LBD* genes is significantly higher in Moso bamboo, consistent with previous reports of a WGD in moso bamboo [22, 41]. We therefore performed intra- and inter-genomic collinearity analyses of the *LBD*s. The moso bamboo genome has 28 pairs of duplicated *LBD* genes, including 26 segmentally duplicated pairs and 2 tandem duplicated pairs and segmental duplicates dominate the expansion of the *LBD* gene family in moso bamboo. Similarly, a previous study reported only three tandem repeat events among 131 *G. hirsutum LBD* genes [25]. Synteny analysis of the moso bamboo genome with four other sequenced plant genomes showed that there was significant collinearity of *LBD* family members between bamboo and the monocots maize and rice. Only a few *LBD* members were collinear between bamboo and the dicots Arabidopsis and pepper. This result is consistent with the evolutionary relationship between dicot and monocot plants.

Although LBD genes have been shown to be downstream genes of a series of transcriptional regulatory networks. However, to date, little research has been done on the regulation of downstream target genes by the TF LBD genes, which induce transcription important in the process of cell dedifferentiation E2Fa, which enhances the formation of healing tissue [42]. In Arabidopsis, LOB/AS2 differentiates stem tips into leaf primordia to form leaves by repressing the expression of KNAT2 and KNAT6 [14, 43]. The GO enrichment results of the identified target genes suggest that PeLBDs may influence multiple regulatory pathways by regulating their target genes. Among the top 20 GO terms, two target genes, *PH02Gene50093* and *PH02Gene50387*, were not only significantly enriched as molecular functions of S-(hydroxymethyl) glutathione dehydrogenase activity but also involved in biological processes such as ethanol metabolism (Supplementary Table 2). Also, significantly enriched were several target genes such as *PH02Gene21014*, *PH02Gene43347* and *PH02Gene43347* associated with the composition of the membrane. These results suggest that target genes regulated by PeLBDs can function through multiple pathways.

Several studies have shown that LBDs regulate lateral organ development and have important effects on plant organs during flower, stem, leaf, and root formation [14, 44, 45], consistent with the results of GO enrichment analysis studies. More than half of the top 20 enriched GO terms in the LBDs were related to plant organ development and formation, among which morphogenic functions involving petal development and leaves were the most significant. *LBD* gene expression profiles were analyzed in different bamboo tissues, and many *PeLBD*s showed relatively high expression levels in specific tissues (Fig. 7). For example, *PeLBD12* and *PeLBD33* were highly expressed in roots, and their functions may be similar to that of *AS2*, which participates directly in the differentiation of stem tip meristematic tissues into leaf primordia in rice, a closely related species. Some *PeLBD* genes were minimally expressed in all the tissues tested, suggesting that they may function in other tissues or at other developmental stages. Interestingly, we found that two genes (*PeLBD20* and *PeLBD29*) were not only highly expressed in the four tissue organs but also detected a consistently elevated expression trend in 0.2–6 m shoots (Fig. 10). This result suggests that *PeLBD20* and *PeLBD29* play an important role in rapid bamboo development. The qRT–PCR was used to further validate the expression of 12 *PeLBD* genes that were expressed at various levels in roots, rhizomes, panicles, and leaves. Among them, we validated a leaf-specific gene, *PeLBD33*, and a flower-specific gene, *PeLBD25*. Overall, the qRT–PCR results differed only slightly from those of the transcriptome sequencing analysis, perhaps due to variations in experimental conditions. Based on these results, we speculate that the *PeLBD* genes play a key role in the tissue development of bamboo.

Early studies on the *LBD* gene family focused on the biological role of these genes in the development of lateral organs in plants. For example, *AtLOB*/*AtASL4* was first shown to be specifically expressed at the base of the proximal axis of lateral organs and at the base of lateral roots in Arabidopsis [6]. The LBD gene family, together with a variety of TFs, forms multiple molecular regulatory networks that are important for plant response to environmental stress and regulation of growth and development and other physiological processes [46]. After stimulation of healing tissue cells by growth hormone signals, lysine methyltransferase (ATXR2) promotes cells to enter the dedifferentiation stage. Subsequently, the TFs ARF7 and ARF19 recruit ATXR2, which in turn combines with the promoter of LBD genes to induce the expression of genes *LBD16*, *LBD17*, *LBD18* and *LBD29* during cell dedifferentiation. LBD genes then regulate E2Fa, an important TF in cell dedifferentiation, thereby enhancing the formation of healing tissue [42]. In this study, we focused on the expression of *PeLBD* genes during the rapid growth of bamboo shoots. We performed temporal clustering analysis using gene Transcriptome data and identified six genes (*PeLBD20*, *PeLBD29*, *PeLBD46*, *PeLBD10*, *PeLBD38*, and *PeLBD06*) whose expression was strongly positively associated with the rapid growth and development of bamboo shoots. The expression levels of these six genes were verified by qRT–PCR (Fig. 10). Their expression tended to increase as the lignification of bamboo shoots increased during growth, with *PeLBD20* and *PeLBD38* showing the most significant positive correlation. It is hypothesized that they play important biological functions in the rapid growth stage of moso bamboo. These results not only strongly suggested the involvement of *LBD*s in the lignification process during rapid growth of bamboo shoots but also provided potential candidate genes for future research on bamboo growth and development.

## Conclusions

This is the first systematic identification and analysis of LBD TFs in Moso bamboo. We identified a total of 55 PeLBD genes, which can be classified into two classes and seven subclasses. Each subclass has similar gene structure and sequence, indicating that LBD genes are conserved during evolution. Evolutionary analyses indicated that segmental duplications associated with WGD events were responsible for most of the expansion of the Mauve LBD gene family. Based on the STEM version software and RNA-seq data, the tissue and temporal specificity of PeLBD gene expression was revealed, which was also supported by quantitative analysis and functional enrichment. Six genes (*PeLBD6/10/20/29/38/46*) showed a continuous upward trend in shoot development, which implies that these genes are vitally important for the fast-growing development of moso bamboo. This study provides a good data base for the in-depth study of the functions of the moso bamboo LBD TF family genes and provides new insights to explore the molecular mechanisms of rapid growth in moso bamboo.

## Methods

### Identification and sequence analysis of LBD proteins from Moso bamboo

Genomic data of moso bamboo was downloaded from the *P. edulis* genome database (ftp://parrot.genomics.cn/gigadb/pub/10.5524/100001_101000/100498/). A hidden Markov model of the lateral organ boundaries domain (DUF260, PF03195) was obtained from the Pfam database (http://pfam.xfam.org/) and used as the seed model for an HMMER3 search (http://hmmer.janelia.org/) of the local bamboo protein database (*E* ≤ 10^− 20^) [47], and redundant genes were removed to produce a set of preliminary LBD candidate sequences. To verify that these candidates are LBDs, we used the SMART (http://smart.embl-heidelberg.de/) [48] and Pfam [49] databases to filter out sequences that lacked a complete LOB domain. The confirmed *LBD* genes were renamed according to their positions on the moso bamboo chromosomes.

Subcellular localization predictions were generated with Cell-PLoc 2.0 (http://www.csbio.sjtu.edu.cn/bioinf/Cell-PLoc-2/) [50], and the ExPASy ProtParam tool (https://web.expasy.org/protparam/) [51] was used to predict protein physicochemical parameters such as molecular weight (MW), isoelectric point (PI) and grand average of hydropathicity (GRAVY).

### Sequence alignment and phylogenetic tree construction

Whole genome information for Arabidopsis and rice was downloaded from the TAIR10 database (http://www.arabidopsis.org/index.jsp) and the Rice Genome Annotation Project database (http://rice.plantbiology.msu.edu), respectively. Maize and pepper genomic data were downloaded from the Ensembl database (http://asia.ensembl.org/index.html).

Forty-three Arabidopsis LBD proteins and 36 rice LBD proteins were identified from HMMER3 searches of the corresponding local protein databases [47]. The Arabidopsis and rice LBD sequences were combined with those from moso bamboo, and a multiple protein sequence alignment was produced with MUSCLE [52]. The resulting alignment was used to construct a maximum likelihood (ML) phylogenetic tree in MEGA 7.0 with 1000 bootstrap replicates [53]. Intraspecific phylogenetic trees were also constructed using the LBD protein sequences from bamboo. The amino acid sequences of conserved domains were compared and edited using Jalview software (http://www.jalview.org/) [54], and conserved motif Logos were generated with the WebLogo program (http://weblogo.threeplusone.com) [55].

### Gene structure, motif composition, and promoter element analysis

The intron–exon distributions of the moso bamboo *LBD* genes were obtained using GFF annotation files from the moso bamboo genome. Conserved amino acid sequences of LBD proteins were analyzed using the online MEME tool (http://meme-suite.org/) [56]. MEME analysis parameters included a minimum width ≥ 6, a maximum width of 50, and a motif number of 6; all other parameters were set to default values. PlantCARE (http://bioinformatics.psb.ugent.be/webtools/plantcare/html/) was used to identify *cis*-acting elements in the 1500-bp promoter region upstream of each gene’s transcription start site, and the results were visualized using TBtools (v1.0697) [57].

### Synteny analysis and Ka/Ks ratios

The moso bamboo protein sequences were aligned to one another or to the protein sequences from Arabidopsis, rice, maize, or pepper using TBtools software. MCScanX [58] was used to identify gene duplication events and syntenic relationships among the LBD proteins, and the results were visualized using Circos and Dual Synteny Plot in TBtools [59].

For Ka/Ks analysis, seventeen homologous gene pairs were identified by BLASTn using two criteria: (1) > 75% sequence similarity and (2) an alignable region > 75% of the length of the longer sequence [60]. KaKs_Calculator2.0 was used to calculate the synonymous substitution rate (Ks), nonsynonymous substitution rate (Ka), and Ka/Ks ratio between homologous gene pairs [61]. Evolutionary divergence times within the bamboo *LBD* gene family were calculated using the bamboo-specific divergence time formula T = Ks/2λ (where λ = 6.5 × 10^− 9^).

### Transcription profiling of *PeLBD*s based on RNA-Seq data

In a recent study, we obtained 24 original transcriptome datasets (accession number GSM2810849: SRR6171235-SRR6171258) from the Gene Expression Omnibus (GEO) database (http://www.ncbi.nlm.nih.gov/geo). Tissue samples from eight fast-growing developmental nodes were included (located at 0.2, 0.5, 1, 2, 3, 4, 5, and 6 m on young shoots), with three biological replicates from each developmental node. In addition, transcriptome data from different plant tissues (roots, rhizomes, panicles, and leaves) were downloaded from the European Nucleotide Archive (https://www.ebi.ac.uk/ena/browser/home) (accession numbers ERR105067–ERR105076). Transcriptome data from different tissues were expressed as log_2_ (TPM + 1) and visualized using the function pheatmap package in R. (https://cran.r-project.org/web/packages/pheatmap/index.html).

The STEM version 1.3.11 [62] was used to analyze and visualize trends in *PeLBD* gene expression during rapid shoot growth with the STEM clustering method, a maximum of 20 model profiles, and all other parameters set to their default values [63].

### Plant material, RNA extraction, and qRT–PCR analysis

The qRT–PCR analysis was used to verify the expression of *PeLBD* genes in different tissues and organs (root, rhizome, panicle and leaf), as well as their expression and potential regulatory roles during the rapid development of bamboo shoots. Bamboo shoots of different heights (0.2 m, 0.5 m, 1 m, 2 m, 3 m, 4 m, 5 m, 6 m) were taken from the part that was combined with the rhizomes, and the underground part was taken from 0.2 m as the base point.

Tissue samples of bamboo rhizomes, inflorescences, young leaves, roots, and shoots of different heights (0.2, 0.5, 1, 2, 3, 4, 5, and 6 m) were obtained from bamboo plants growing in a Bamboo Garden, Guilin city, Guangxi, China. The study area is 155 m above sea level, an average annual temperature is 19.0 °C | 66.3 °F, and the mean annual rainfall is 2174 mm | 85.6 in.. All materials collected in the field did not require ethical approval and a license. Three biological replicates of each tissue type were obtained. After harvesting, tissue samples were immediately placed in liquid nitrogen and stored at − 80 °C until RNA extraction.

Total RNA was extracted from each sample using the FastPure Plant Total RNA Isolation kit (Nanjing Vazyme Biotech, China, RC401), and the reverse-transcribed cDNA products were stored at − 20 °C for backup. cDNA was diluted five-fold before using as a template. Primers for qRT–PCR were designed using Beacon Designer 7 (Supplemental Table 1). The qRT–PCR reaction system (final volume 20 μL) contained 10 μL 2 × chamQ Universal SYBR qPCR Master Mix, 0.4 μL 10 μM forward primer, 0.4 μL 10 μM reverse primer, 2 μL template cDNA, and 7.2 μL ddH_2_O. The reaction program was 95 °C for 30 s; 95 °C for 10 s and 60 °C for 30 s; and 40 cycles of 95 °C for 15 s, 60 °C for 60 s, and 95 °C for 15 s. There were three technical replicates per sample. *PeActin* (*PH02Gene08372*) was used as the internal reference gene. Amplification was performed using a Bio-Rad iCycler iQ real-time quantitative PCR instrument (CFX96, USA), and the relative expression level of each gene was calculated using the 2^−ΔΔCt^ method. All statistical analysis was performed using GraphPad Prism 7 Software. Comparisons between paired groups were performed with Student’s t-test.

### Protein–protein interaction (PPI) network construction and GO enrichment analyses

The LBD protein sequences were uploaded to the STRING database (https://string-db.org/) for node comparison, and relationships among important proteins were predicted based on rice protein interactions. Cytoscape (V3.7.1) was used to visualize the resulting network [64].

GOATOOLS (http://github.com/tanghaibao/GOatools) [65] was used to assign Gene Ontology (GO) annotations to LBDs, and Fisher’s exact test was used to identify biological functions enriched in the PeLBDs relative to the full GO database. An false discovery rate (FDR) multiple testing correction [66] was used to minimize false positives, and functions were considered to be significantly enriched when their FDR-corrected *P*-values (Padjust) were < 0.05.

### Identification and annotation of LBD target genes

To obtain a list of downstream target genes potentially regulated by the LBDs, we used TBtools (v1.0697) [59] to extract the 2000-bp promoter sequences of the moso bamboo genes. The consensus motif of the LBD DNA binding site (MA1673.1) was obtained from the JASPA_CORE database (http://jaspar.genereg.net) of eukaryotic TF binding profiles [67]. The Motif FIMO program in the MEME suite (5.3.0) (http://meme-suite.org/) [56] was then used to detect the consensus LBD binding motif in the moso bamboo promoter set. Final target gene candidates were identified based on a screening criterion of *P* < 1 × e^− 6^. The candidate LBD target genes were functionally annotated using the GO and Kyoto Encyclopedia of Genes and Genomes (KEGG) databases. Analysis and visualization were performed using the Majorbio online platform (https://cloud.majorbio.com).

## Supplementary Information

**Additional file 1: Supplemental Fig. 1.** The total number of LBD gene subfamilies in the nine species.

**Additional file 2: Supplemental Fig. 2.** The LOGO of six amino acid motifs in LBD proteins.

**Additional file 3: Supplemental Fig. 3.** Number of cis–acting elements on promoters of PeLBD genes.

**Additional file 4: Supplemental Fig. 4.** The consensus motif of the LBD DNA binding site from the JASPA_CORE database.

**Additional file 5: Supplemental Fig. 5.** GO analysis of potential PeLBD target genes.

**Additional file 6: Supplemental Fig. 6.** KEGG analysis of potential PeLBD target genes.

**Additional file 7: Supplemental Table 1.** The Ka-Ks analysis of 17 PeLBD duplicated gene pairs.

**Additional file 8: Supplemental Table 2.** Detailed information on GO and KEGG enrichment analysis in PeLBD target gene.

**Additional file 9: Supplemental Data 1.** Detailed information on interspecies synteny of the moso bamboo genome.

**Additional file 10: Supplemental Data 2.** The specific primers of PeLBD genes for qRT-PCR.

**Additional file 11: Supplemental Data 3.** Detailed information on gene ontology enrichment analysis in PeLBD family.

## Data Availability

All data generated or analysed during this study are included in this published article and its supplementary information files. The raw sequencing data used during this study have been deposited in NCBI (http://www.ncbi.nlm.nih.gov/geo) and European Nucleotide Archive (https://www.ebi.ac.uk/ena/browser/home) under accession numbers GSM2810849: SRR6171235-SRR6171258 and ERR105067-ERR105076, respectively. Public access to the library is completely open.

## Abbreviations

MCScanX: Multiple Collinearity Scan toolkit
Ka: Non-synonymous
Ks: Synonymous
TPM: Transcripts Per Kilobase Million
WGDs: whole genome duplications
STEM: Short Time-series Expression Miner
KEGG: Kyoto Encyclopedia of Genes and Genomes databases
