# Supplementary material for: Genome-wide identification and expression analysis of LBD transcription factor genes in Moso bamboo (Phyllostachys edulis)
Source: BMC Plant Biol. 2021 Jun 28;21:296. doi: 10.1186/s12870-021-03078-3 (PMC8240294; doi:10.1186/s12870-021-03078-3)
Supplement: Supplementary file 1 — Additional file 1: Supplemental Fig. 1. The total number of LBD gene subfamilies in the nine species. [file 12870_2021_3078_MOESM1_ESM.pdf]

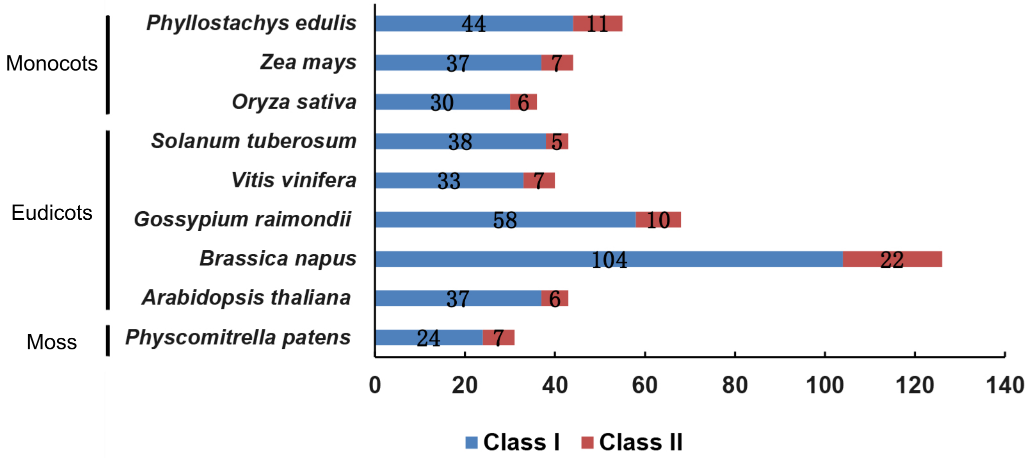
Monocots
Eudicots
Moss
Phyllostachys edulis
Zea mays
Oryza sativa
Solanum tuberosum
Vitis vinifera
Gossypium raimondii
Brassica napus
Arabidopsis thaliana
Physcomitrella patens
44
11
37
7
30
6
38
5
33
7
58
10
104
22
37
6
24
7
0
20
40
60
80
100
120
140
Class I
Class II
