# Supplementary figures and images for: Genome-wide identification and expression analysis of LBD transcription factor genes in Moso bamboo (Phyllostachys edulis)
Source: BMC Plant Biol. 2021 Jun 28;21:296. doi: 10.1186/s12870-021-03078-3 (PMC8240294; doi:10.1186/s12870-021-03078-3)

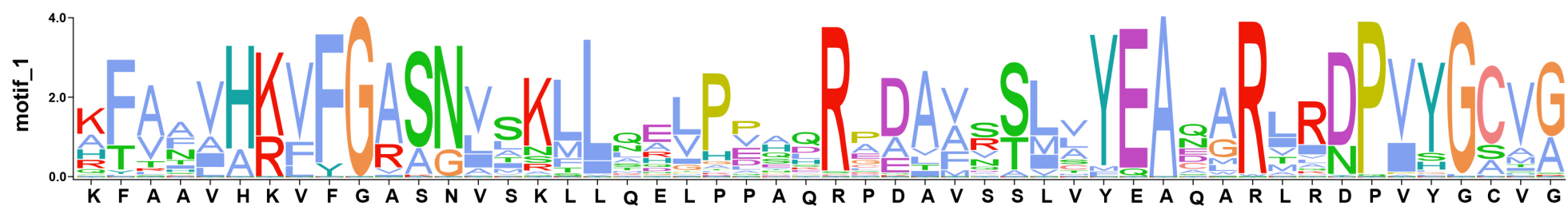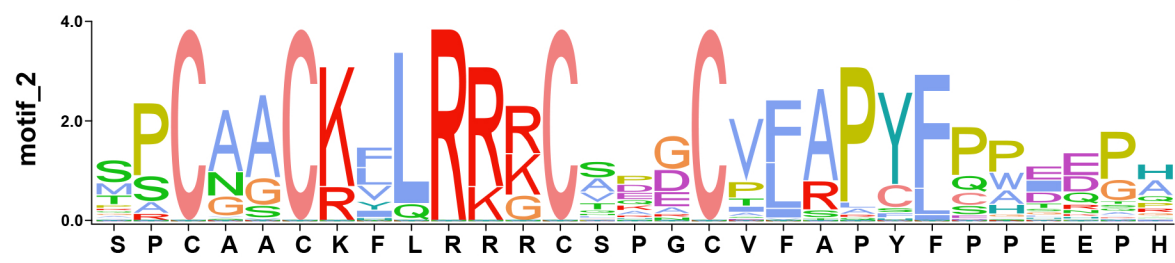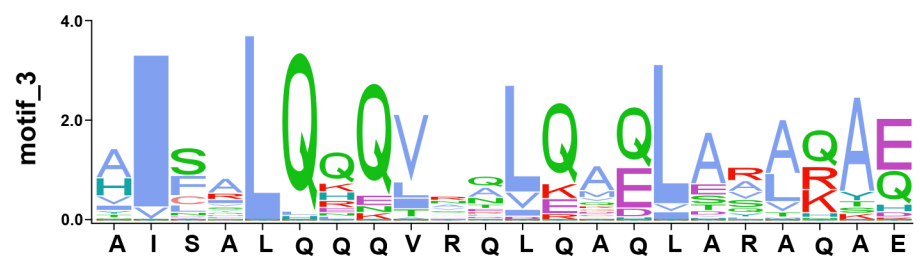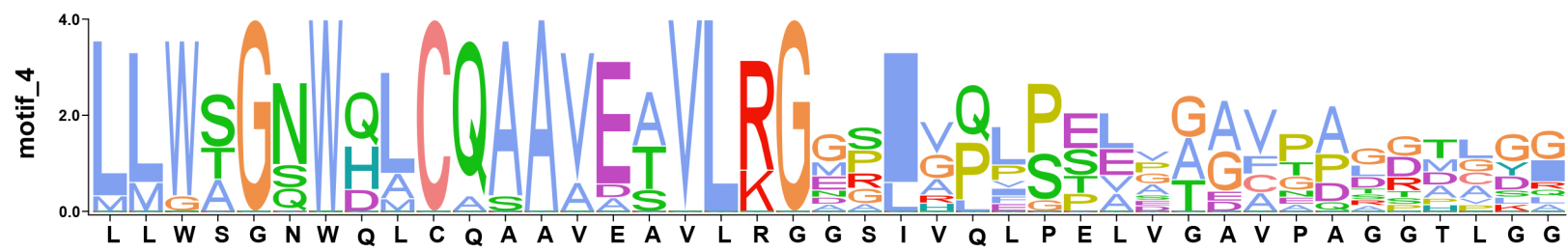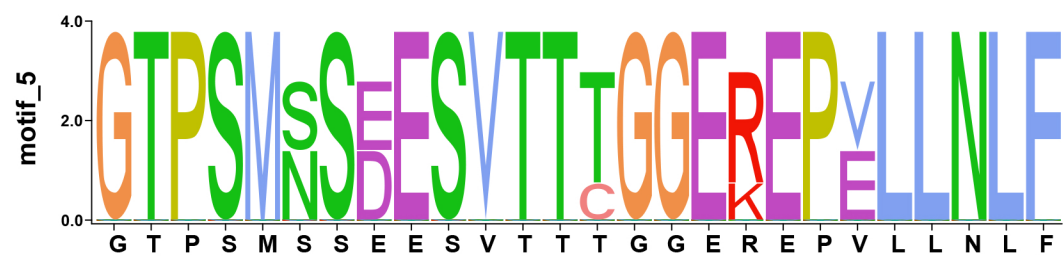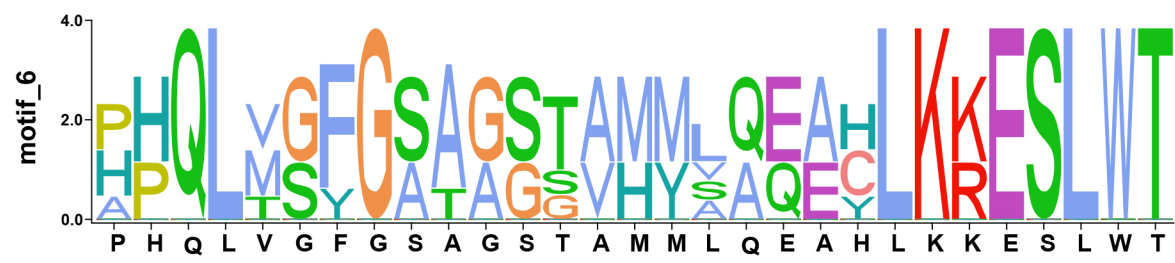

Supplement: Supplementary file 2 — Additional file 2: Supplemental Fig. 2. The LOGO of six amino acid motifs in LBD proteins. [file 12870_2021_3078_MOESM2_ESM.pdf]

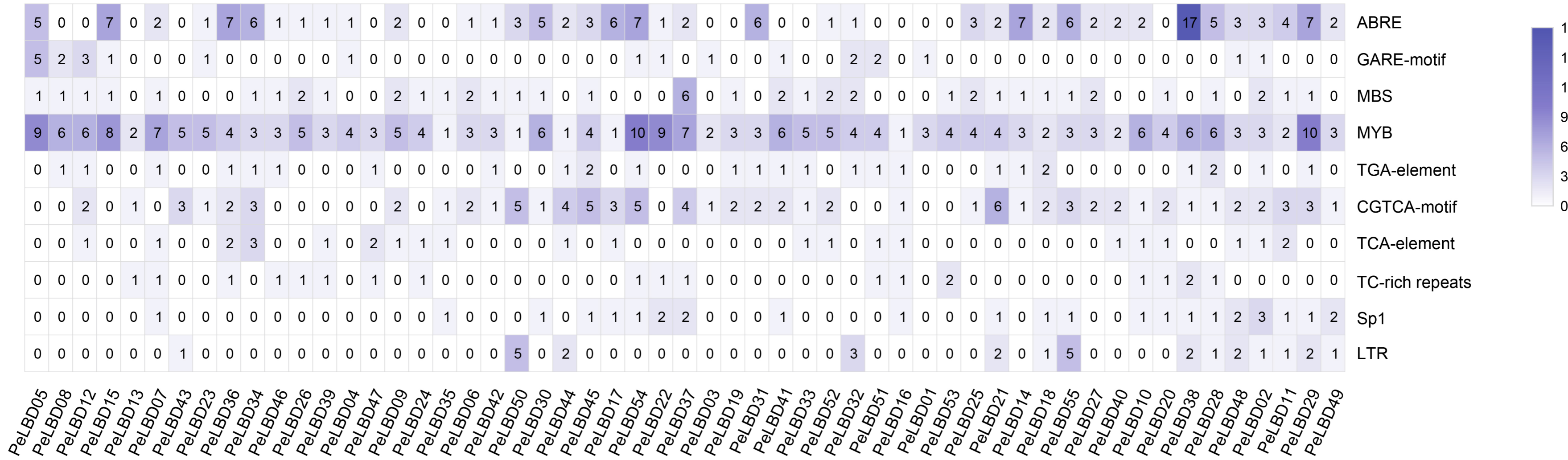

Supplement: Supplementary file 3 — Additional file 3: Supplemental Fig. 3. Number of cis–acting elements on promoters of PeLBD genes. [file 12870_2021_3078_MOESM3_ESM.pdf]

# Consensus LBD

Bits

2.0

1.5

1.0

0.5

0.0

1

2

3

4

5

6

7

8

9

10

11

12

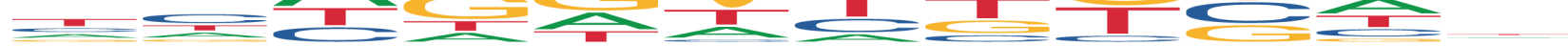

Supplement: Supplementary file 4 — Additional file 4: Supplemental Fig. 4. The consensus motif of the LBD DNA binding site from the JASPA_CORE database. [file 12870_2021_3078_MOESM4_ESM.pdf]

## GO Classification

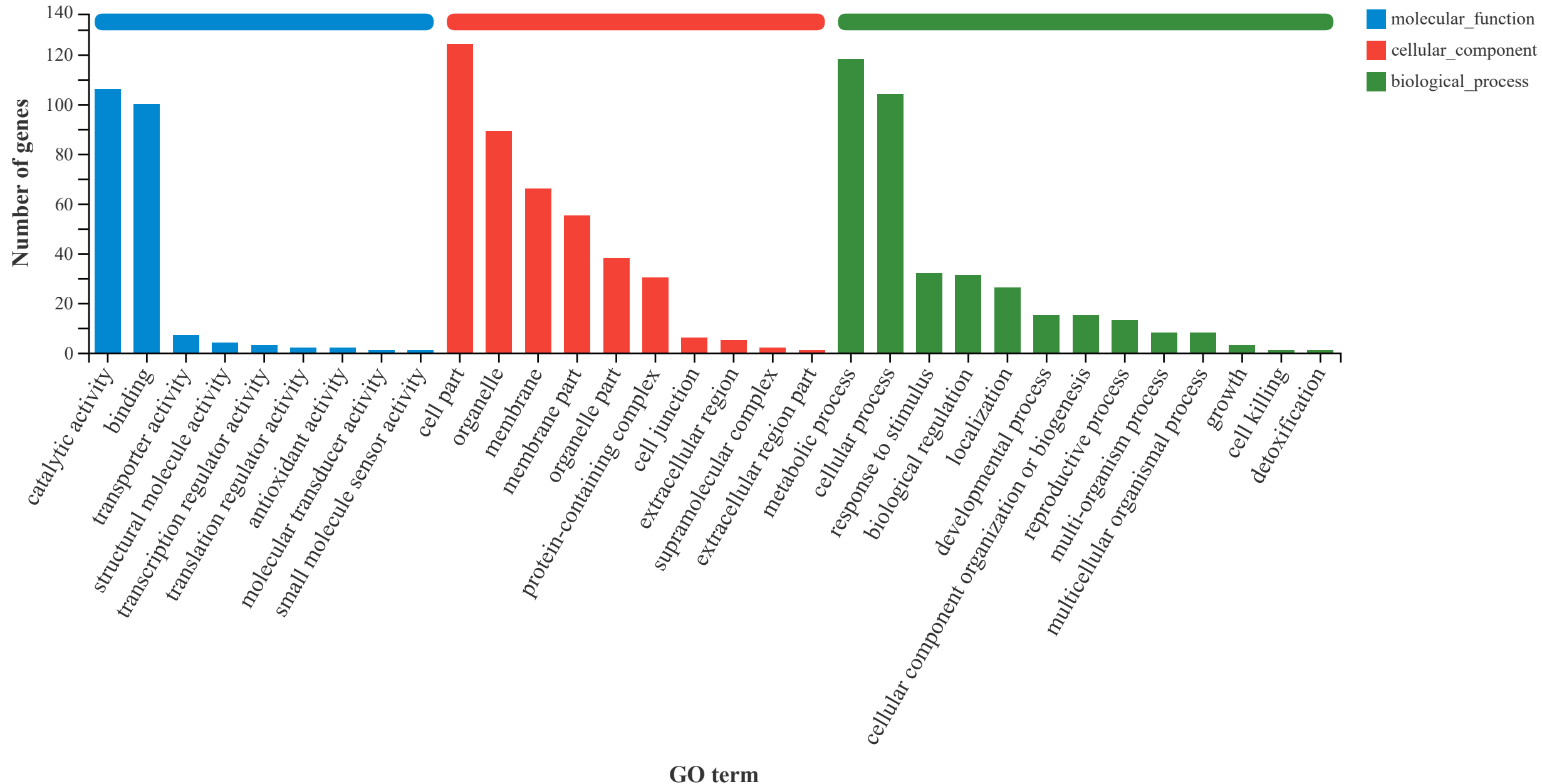

Supplement: Supplementary file 5 — Additional file 5: Supplemental Fig. 5. GO analysis of potential PeLBD target genes. [file 12870_2021_3078_MOESM5_ESM.pdf]
