# Supplementary material for: Genome-wide identification and expression analysis of LBD transcription factor genes in Moso bamboo (Phyllostachys edulis)
Source: BMC Plant Biol. 2021 Jun 28;21:296. doi: 10.1186/s12870-021-03078-3 (PMC8240294; doi:10.1186/s12870-021-03078-3)
Supplement: Supplementary file 6 — Additional file 6: Supplemental Fig. 6. KEGG analysis of potential PeLBD target genes. [file 12870_2021_3078_MOESM6_ESM.pdf]

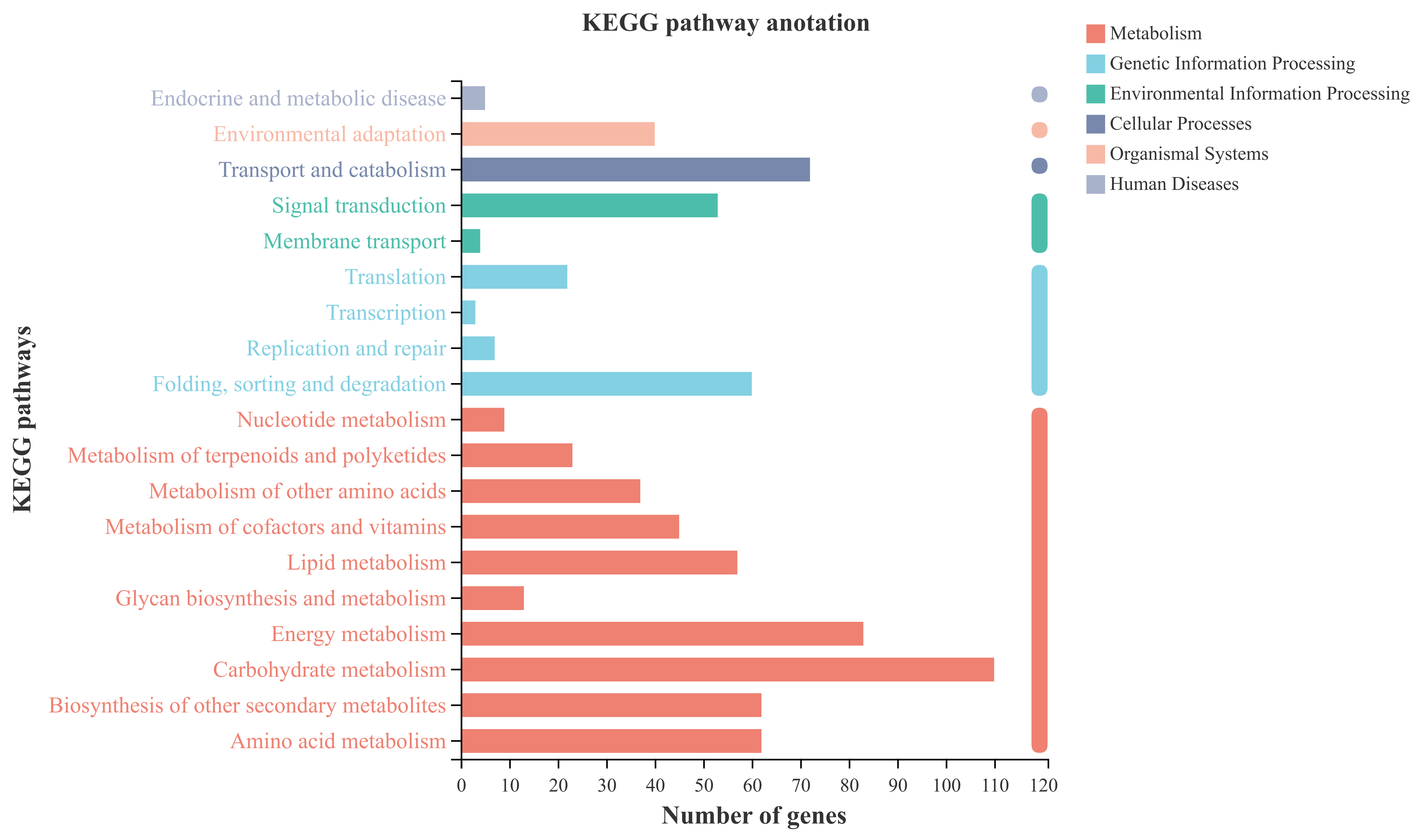
KEGG pathway anotation
KEGG pathways
Endocrine and metabolic disease
Environmental adaptation
Transport and catabolism
Signal transduction
Membrane transport
Translation
Transcription
Replication and repair
Folding, sorting and degradation
Nucleotide metabolism
Metabolism of terpenoids and polyketides
Metabolism of other amino acids
Metabolism of cofactors and vitamins
Lipid metabolism
Glycan biosynthesis and metabolism
Energy metabolism
Carbohydrate metabolism
Biosynthesis of other secondary metabolites
Amino acid metabolism
0 10 20 30 40 50 60 70 80 90 100 110 120
Number of genes
Metabolism
Genetic Information Processing
Environmental Information Processing
Cellular Processes
Organismal Systems
Human Diseases
